# Binding modes of a flexible ruthenium polypyridyl complex to DNA†

**DOI:** 10.1039/d4cp02782e

**Published:** 2024-10-11

**Authors:** Meritxell Malagarriga, Leticia González

**Affiliations:** a Institute of Theoretical Chemistry, Faculty of Chemistry, University of Vienna, Währinger Straße 17 1090 Vienna Austria; b Doctoral School in Chemistry (DoSChem), University of Vienna, Währinger Straße 42 1090 Vienna Austria; c Vienna Research Platform on Accelerating Photoreaction Discovery, University of Vienna, Währinger Straße 17 1090 Vienna Austria leticia.gonzalez@univie.ac.at

## Abstract

Ruthenium(ii) polypyridyl complexes are attractive binders to DNA. Modifying the hydrophobicity, shape, or size of the ancillary ligands around the central ruthenium atom can induce changes in the binding mode to the DNA double helix. In this paper, we investigate the binding modes of [Ru(2,2′-bipyridine)_2_ (5-{4-[(pyren-1-yl)methyl]-1*H*-1,2,3-triazol-4-yl}-1,10-phenanthroline)]^2+^ (RuPy for short), a metal complex featuring a flexible pyrene moiety known for its intercalative properties. Classical molecular dynamics simulations are employed to gain insight into the non-covalent binding interactions of RuPy with two different 20 base pair DNA sequences, poly(dA)poly(dT) (AT) and poly(dC)poly(dG) (CG). In addition to examining the intercalation of the pyrene moiety from the major groove, the stability of RuPy–DNA adducts is investigated when the metal complex interacts externally with the DNA and with the major and minor groove pockets. The results indicate that external interaction and major groove binding are not stable, whereas intercalation consistently forms stable adducts. Minor groove binding showed less stability than intercalation and more variability, with some trajectories transitioning to intercalation, involving either the pyrene moiety or a bipyridine ligand. Pyrene intercalation, especially from the minor groove, was the most stable, while bipyridine intercalation was less favorable and associated with higher binding free energies.

## Abstract 2

Ruthenium(ii) polypyridyl complexes are attractive binders to DNA.

## Graphical abstract

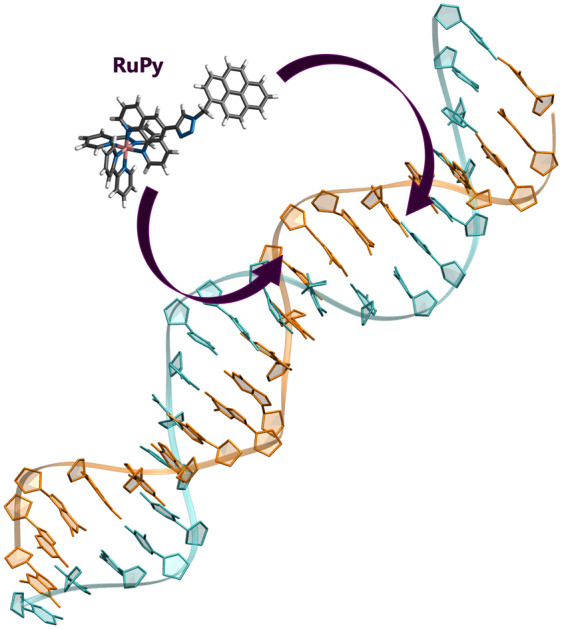

## 1 Introduction

Recent years have seen extensive research on a variety of ruthenium transition metal complexes due to their remarkable photophysical and photochemical properties, which are determined by the configurations of their ancillary ligands.^1–5^ Ruthenium(ii) polypyridyl complexes are of particular interest owing to their potential as light harvesting photosensitizers and electron or charge transfer agents^6–9^ as well as their broad applicability in biomedical applications, such as DNA imaging,^10^ photodynamic therapy,^11–13^ anticancer therapeutics,^14,15^ or structural probes.^16^ For the latter, it is convenient that the Ru complex is able to interact non-covalently with organic molecules in a kinetically inert fashion such as with nucleic acids^17,18^ and specifically with double-stranded DNA.

The interaction of ruthenium complexes with DNA can trigger a change on its photophysical properties. The light-switch effect, *i.e.* the fact that the complex emits minimally in aqueous solution but luminescence is highly enhanced when the complex interacts with DNA—as first reported by Barton and co-workers with the [Ru(bpy)_2_ (dppz)]^2+^ complex—is a good example.^19^ It is the planarity and the extended π-conjugated character of the dppz ligand that makes the complex bind non-covalently in an intercalative manner between adjacent nucleobase pairs of the DNA double strand, preventing the protic solvent molecules from quenching luminescence.^20,21^

Besides intercalative binding, which is stabilized by π–π stacking interactions between planar aromatic moieties placed within nucleobase pairs, alternative non-covalent binding modes can take place between double-stranded DNA and ligands of the ruthenium-based complexes.^16,22^ Being positively charged, ruthenium(ii) polypyridyl complexes can externally interact with the negatively charged phosphate backbone of DNA *via* electrostatic effects. Additionally, they can also interact with the DNA double helix major and minor grooves, mainly but not exclusively *via* van der Waals interactions. Thus, modifying the hydrophobicity, shape or size of the ancillary ligands around the central ruthenium atom can induce a change of the binding mode, affinity and selectivity to the DNA double helix, which ultimately also changes the photochemical behavior of the complex. For this reason, substantial effort has been devoted into tuning the coordination sphere of Ru with the hope to discover new DNA probes or influence different chemical processes within the DNA polymer.^23–25^ Interestingly, while the vast majority of studies involve large rigid aromatic ligands, as an attempt to enhance the luminescence with respect to [Ru(bpy)_2_ (dppz)]^2+^, there seems to be less research on ruthenium(ii) polypyridyl complexes containing flexible ligands.

**Fig. 1:**
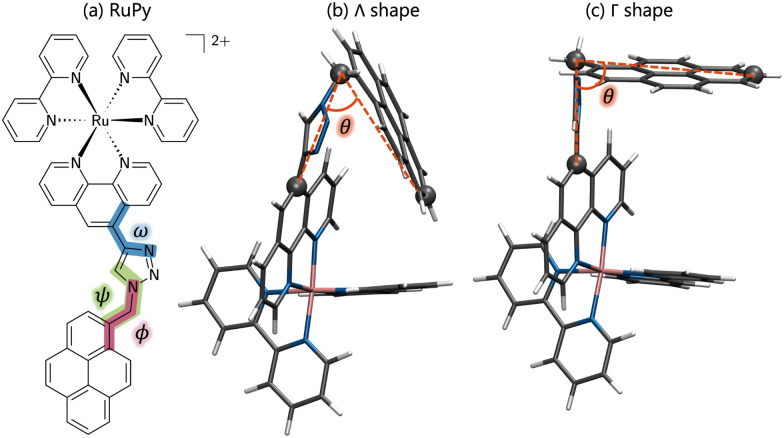
(a) Structure of the ruthenium(ii) complex (RuPy) depicting the *ϕ*, *ω* and *ψ* torsional angles in red, blue and green, respectively. (b) RuPy in the Λ-shaped geometry, and (c) Γ-shaped geometry. Color code for atoms and angle *θ*: grey for C, blue for N, pink for Ru, white for H, and red for *θ*.

In this work we are interested in [Ru(2,2′-bipyridine)_2_(5-{4-[(pyren-1-yl)methyl]-1*H*-1,2,3-triazol-4-yl}-1,10-phenanthroline)]^2+^ (RuPy for short, see Fig. 1) as a photosensitizer to be employed for photocatalysis. Being highly hydrophobic, pyrene is known to intercalate within DNA base pairs.^26–28^ Thus, RuPy, with its flexible pyrene moiety, is also expected to readily interact with a DNA double helix, and thus remained anchored for electron injection into a catalyst after light excitation. As a first step in using RuPy as a photosensitizer for photocatalysis, the aim of this paper is to unravel by means of using classical molecular dynamics (MD) simulations the possible binding modes of RuPy to DNA. A thorough understanding of how RuPy binds to DNA also contributes to the broader knowledge of DNA interactions with metal complexes and can help to understand biological implications related to potential therapeutic or toxic effects.

## 2 Computational methods

### 2.1 Generation of initial structures

**Fig. 2:**
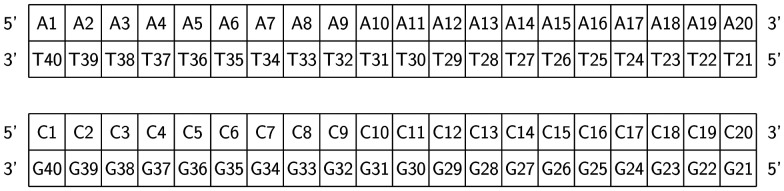
Schematic representation of the AT and CG sequences of the two oligonucleotides employed in this work.

RuPy has been non-covalently bound to the two different 20-mer DNA duplex sequences, shown in Fig. 2. One is formed from a single strand of adenine (A) and the complementary thymine (T) nucleobases (poly(dA)poly(dT) or AT for short). The other is constituted by one single strand of cytosine (C) nucleobases and the complementary guanine (G) nucleobases (poly(dC)poly(dG), and referred as CG). The two initial icosamers were created using the nucleic acid builder module in AmberTools23.^29^

The initial structure of RuPy was built using GaussView^30^ and then optimized at the B3LYP/def2-SVP level of theory employing the Grimme's D3 model with Becke–Johnson damping (D3(BJ))^31^ to empirically correct for dispersion interaction effects, as in ref. 32–34. The optimization was done in water using the implicit conductor-like polarizable continuum model.^35^ A frequency calculation at the same level of theory was done to confirm that the optimized geometry was as a minimum by the absence of imaginary frequencies within the harmonic approximation. These calculations were done using the Gaussian16^36^ software.

Due to the flexibility of the pyrene ligand, an extensive conformational analysis was mandatory to identify initial geometries prior the interaction with DNA. The geometries were characterized by the three torsional angles *ϕ*, *ω* and *ψ*, shown in Fig. 1a. Conformational ensembles were generated in the gas phase using the conformer-rotamer ensemble sampling tool (CREST).^37–39^ For comparison, both the extended semiempirical tight-binding (xTB)^40,41^ and the generic polarizable force-field of the geometries, frequencies, and non-covalent interaction (GFN) family of methods GFN-FF levels of theory were employed. In order to discard geometries lying high in energy, pruning was done by performing single point energy calculations on all the structures at the B3LYP-D3(BJ)/def2-SVP level of theory and taking into account implicit water solvent effects. As suggested elsewhere,^42^ the threshold was set to 30 kJ mol^−1^ of energy difference between the most stable structure and any other, obtained at the xTB or GFNFF level of theory within CREST. The structures lying within the relevant energy range were subsequently reoptimized at the B3LYP-D3(BJ)/def2-SVP level of theory. The Conformer-Rotamer Ensemble GENeration (CREGEN) algorithm implemented in CREST was used to sort the different conformations into ensembles based on the energy, rotational constants, and Cartesian root mean squared deviations (RMSDs). Representatives of the most stable clusters were identified and categorized as Λ or Γ (Λ-shaped and Γ-shaped) depending on the relative position of the pyrene moiety with respect to the rest of RuPy, characterized by the angle *θ* (see Fig. 1b). Geometries with *θ* values below 70 were labeled as Λ-shaped and *θ* values around 90 as Γ-shaped geometries. Λ-Shaped geometries were not expected to interact favorably with DNA, yet the most stable form was selected for subsequent RuPy/DNA interaction studies, together with the four most stable Γ-shaped geometries.

**Fig. 3:**
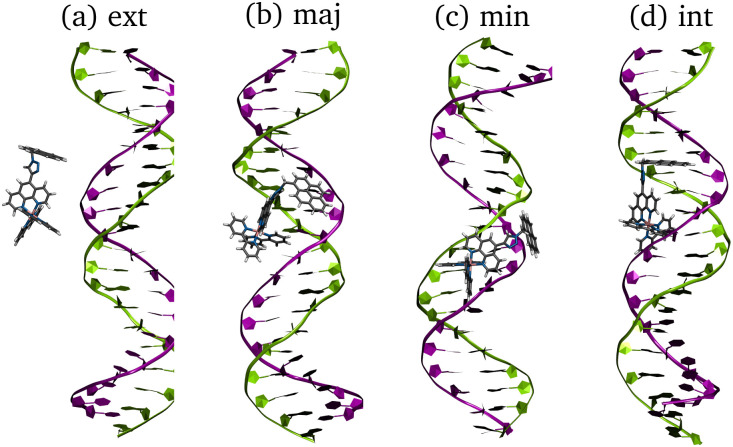
Initial structures of Γ_0_-RuPy/AT-DNA complexes in (a) external (ext) interaction, (b) major groove (maj), (c) minor groove (min) and (d) intercalation (int) binding modes. Color code for atoms in RuPy and residues in AT: grey for C, blue for N, pink for Ru, white for H, green for adenine, and purple for thymine.

To check for possible spontaneous associations also an ensemble of non-interacting RuPy-DNA systems was set up. However, the simulation times proved not to be enough to simulate spontaneous intercalation (see Section S2 in the ESI†). Therefore, the five selected RuPy structures (Λ_0_-RuPy and Γ_*i*_-RuPy with *I* = 0–3) were non-covalently bonded to the two AT and CG sequences. Initially, Λ_0_-RuPy was manually docked in four different binding sites: externally (ext), in the major groove (maj), minor groove (min), and the most stable Γ-shaped RuPy structure (coined Γ_0_-RuPy) was also manually intercalated (int), as depicted in Fig. 3. While bpy ligand can interact with DNA, it lacks the extended planar structure that characterizes full intercalators; therefore, it has not been considered as a initial binding pose. For groove binding, the DNA structures and RuPy were relatively oriented such that the pyrene moiety of RuPy faces towards the DNA. Since aromatic planar units are known to intercalate,^26,43^ for the intercalative binding, the pyrene moiety was manually inserted between the the base pair steps 5′-A-T-3′ and 5′-C-G-3′ in the AT and CG sequences, respectively, through the major groove and such that the pyrene unit was perpendicular to the helical axis of DNA. In view that the trajectories did not converged for Γ_0_-RuPy in external and major groove binding, for the remaining three selected Γ-shaped geometries of RuPy (Γ_*i*_-RuPy, *i* = 1–3), only the minor groove and intercalative bindings were considered further. As expected, the selected Λ-shaped geometry (Λ_0_-RuPy) could not be intercalated due to steric hindrance. Therefore, it was only placed in external, major and minor groove binding. Manual docking was chosen over DNA-ligand docking algorithms due to the specific challenges of the latter. Traditional docking methods, often adapted from protein–ligand models, are not suited for DNA's charged environment, polarization effects, or the conformational changes that occur during ligand binding, particularly in intercalation.^44^ These methods assume a rigid receptor, limiting their accuracy for DNA-ligand interactions. Manual docking, guided by prior knowledge of DNA and the RuPy complex,^26,28^ allowed us to position the ligand in plausible binding modes and efficiently prepare the system for MD simulations.

### 2.2 Molecular dynamics simulations

Classical MD simulations were performed using the pmemd engine of the AMBER software.^45^ The AT and CG oligonucleotides were described using the OL21 AMBER force field^46^ included in AmberTools23,^29^ and subsequently neutralized with K^+^ ions. RuPy was described with the Generalized Amber Force Field (GAFF) with additional parameters developed by Brandt *et al.*^47^ and later used by others.^48–50^ The electrostatic potential for RuPy was computed with the Gaussian16^36^ software at the same level of theory used in the optimizations. Antechamber module in AmberTools23^29^ was used to fit the charges using the restrained electrostatic potential (RESP) approach. Positive charges were neutralized with Cl^−^ ions. The LEaP module of AmberTools23^29^ was employed to parameterize all systems. They were all solvated within a periodically octahedral box of water molecules described by the OPC model,^51^ proven to be optimal in combination with the chosen FF for DNA.^52^ The size of the box was set such that all solute atoms laid at least at 15 Å from any box edge.

The protocol for the isolated DNA, the isolated RuPy and the RuPy/DNA adduct simulations started with a system minimization to release excess strain through 5000 steepest descent cycles followed by 5000 conjugate gradient cycles at constant pressure. After minimization, temperature was risen from 0 to 300 K in a 100 ps constant pressure heating stage with 2 fs time step, using Langevin dynamics temperature scaling with collision frequency *γ* = 1.0 ps^−1^. Periodic boundary conditions were applied, with a cutoff for non-bonded interactions of 10.0 Å Afterwards, a 10 ns equilibration trajectory with 2 fs timestep was run at constant pressure and also using Langevin dynamics with collision frequency *γ* = 1.0 ps^−1^ to maintain the system temperature at 300 K. The length of all bonds involving hydrogen atoms were constraint with the SHAKE algorithm.^53^ Periodic boundary conditions were imposed and the cutoff for non-bonded interactions was set to 10.0 Å. Finally, a production trajectory with the same parameters and conditions as the equilibration was conducted. For adducts with Λ-RuPy and Γ_0_-RuPy in all initial binding modes, the production runs were initially 50 ns long. The trajectories exhibiting low RMSD values were extended up to 100 ns. In view of the results regarding stability of the adducts, production runs for Γ_0_-RuPy in intercalative and minor groove binding were finally extended up to 300 ns. The isolated DNA double strand systems, the isolated RuPy, as well as Γ_*i*_-RuPy/DNA (*i* = 1, 2, 3) with the complex in the minor groove and intercalated were set to 300 ns from the outset. Snapshots of the production run were recorded every 100 ps the first 200, and every 200 ps the last 100 ns.

### 2.3 Analysis of trajectories

All trajectories were visualized by means of VMD^54^ and analyzed using the CPPTRAJ^55^ module of AmberTools23.^29^ The later was used to prove the convergence of the box dimensions and analyze convergence of trajectories by computing the RMSD of heavy atoms of both DNA and RuPy against the starting structure. The distances between DNA base pairs, RuPy angle *θ* and torsional angles *ϕ*, *ω*, *ψ*, and number of contacts between the DNA sequences and RuPy were also analyzed using CPPTRAJ. The number of short range contacts (*N*_SRC_) and the number of long range contacts (*N*_LRC_) were computed using an atom-to-atom cutoff of 4 and 8 Å, respectively, as a means of characterizing the binding mode in each particular time of the simulation. *N*_SRC_ provides an indication of the van der Waals interaction energy between RuPy and DNA, which is particularly relevant when the ligand is situated within the major or minor groove pockets. Similarly, *N*_LRC_ can be correlated with the electrostatic interaction energy, which plays a crucial role in the external binding mode. Null values for both *N*_SRC_ and *N*_LRC_ indicate that the RuPy complex has evolved into the bulk solvent.

**Fig. 4:**
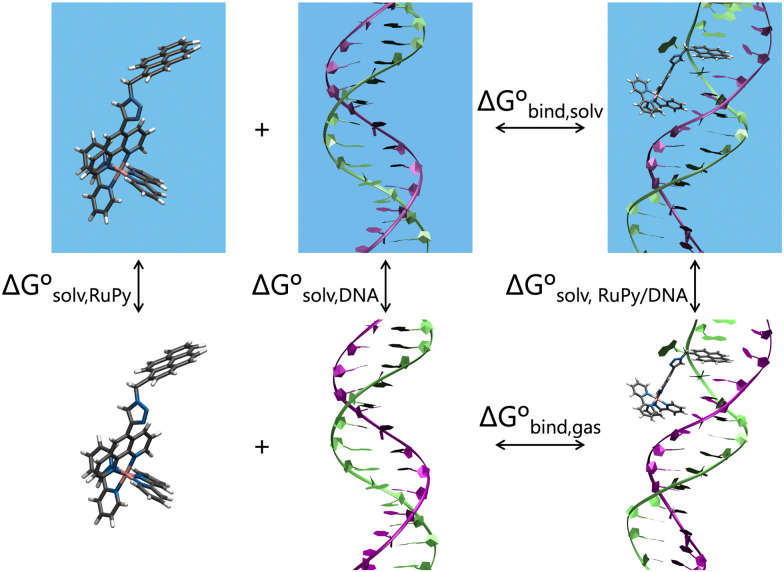
Thermodynamic cycle scheme for binding free energy calculation with the MM-PBSA approach. Systems without boxes depict gas phase, while systems in solvation are shown in blue boxes.

Effective binding free energies were computed for the association of RuPy to the DNA sequences of minor groove and intercalative binding converged trajectories by means of the molecular mechanics Poisson–Boltzmann surface area (MM-PBSA) approach,^56^ where the binding free energy is calculated following the thermodynamic cycle shown in Fig. 4 by subtracting the free energies of the solvated unbound DNA and RuPy from the free energy of the solvated adduct RuPy/DNA

1 Δ*G*_bind,solv_ = Δ*G*_RuPy/DNA_ − (Δ*G*_DNA_ + Δ*G*_RuPy_).

The free energies on the right hand side of eqn (1) are estimated according to

2 Δ*G*_sX_ = *E*_gas,X_ + Δ*G*_solv,X_ − *TS*_X_.

**Fig. 5:**
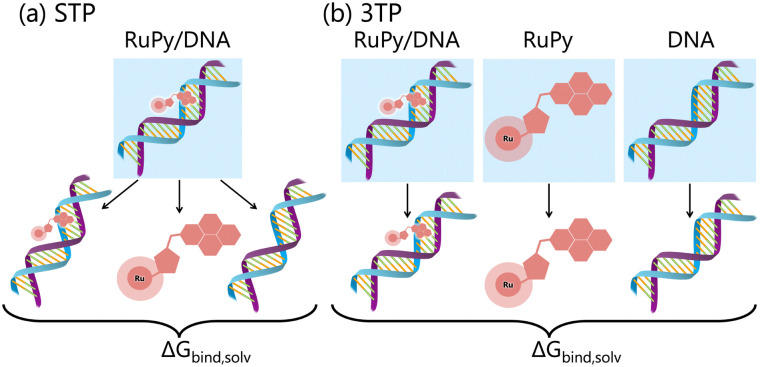
Schematic representation of binding free energy calculation within the MM-PBSA approach with the (a) single trajectory protocol (STP) and (b) three trajectory protocol (3TP).

The gas phase energies *E*_gas,X_ are the molecular mechanical energies from the force field, resulting from considering both bonded and non-bonded (Coulomb and van der Waals) interactions. Solvation effects in Δ*G*_solv,X_ are considered by incorporating two contributions. A polar term is calculated *via* the finite-difference solution of the Poisson–Boltzmann equation as described elsewhere,^57,58^ while a non-polar contribution is estimated based on the solvent accessible surface area.^59^ The solute entropy *S*_X_ can be estimated by either a normal mode analysis or a quasi-harmonic analysis, both of which rely on the rigid-rotor harmonic oscillator approximation.^56^ In this study the entropic term was disregarded since our focus was to obtain relative binding free energies rather than absolute values. Despite MM-PBSA is not accurate to compute absolute free energies, it has been widely used for estimating relative binding energies.^60–62^ Our calculations were done using the MMPBSA.py program within the AmberTools package in AMBER.^29,63^Eqn (1) and (2) refer to single-point energies of the system; however, the MMPBSA.py program calculates binding free energies by averaging the energy contributions from all conformers in a conformational ensemble generated through MD simulations. To generate this ensemble, two different protocols can be utilized (Fig. 5). The single trajectory protocol (STP) derives the bound and unbound states of the ligand and receptor from a single trajectory in which they are already bound. This method is computationally less demanding but does not account for conformational changes induced by binding. Alternatively, the three trajectory protocol (3TP) uses separate trajectories for the ligand, receptor, and adduct to compute the binding free energy. This approach is computationally more expensive but it accounts for the possible conformational changes of both the ligand and receptor due to their interaction. Here, because conformational changes in both the RuPy and DNA double strands were noticed, both protocols were performed for comparison. As we will see, employing both protocols is essential for a comprehensive understanding of the binding interactions in flexible systems like RuPy/DNA adducts. The STP gives us a reliable baseline of the interaction stability, while the 3TP allows us to explore the conformational adaptability and its influence on binding energetics. Together, these approaches provide a more complete picture of the RuPy/DNA interaction landscape, ensuring that we account for both the stability of the binding modes and the flexibility of the interacting partners. A more detailed justification is on Section S3 in the ESI.†

For the STP, the RuPy and DNA geometries were extracted from all snapshots of the RuPy/DNA adducts production runs by stripping water molecules and ions. In two special cases, where the binding mode changed during the simulation, binding free energies were computed for the two binding modes sequentially in time. One case was the conformation Γ_0_-RuPy, which was initially placed in the minor groove pocket of the AT sequence but evolved to intercalated after 50 ns; accordingly, the binding free energy for minor groove binding was computed considering the first 500 snapshots of the production run, while the following 2000 were used to compute the binding free energy for intercalation approaching by the minor groove, since after 200 ns, intercalation ceased. The other case involved the Γ_3_-RuPy/CG adduct, where Γ_3_-RuPy started in minor groove binding but intercalated after 70 ns. Likewise, the first 700 snapshots were employed to calculate the minor groove binding free energy and the remaining were used for intercalative binding. For the 3TP protocol we used the same trajectories as in the STP for the RuPy/DNA adduct. The geometries for isolated RuPy and DNA were extracted from all snapshots of isolated production runs previously computed, also by stripping water molecules and ions. In both protocols, external and internal dielectric constants were set to 80.0 and 1.0, respectively, and ionic strength in molarity was set to zero.

## 3 Results and discussion

### 3.1 Conformational analysis of Ru(ii) complexes in solution

**Fig. 6:**
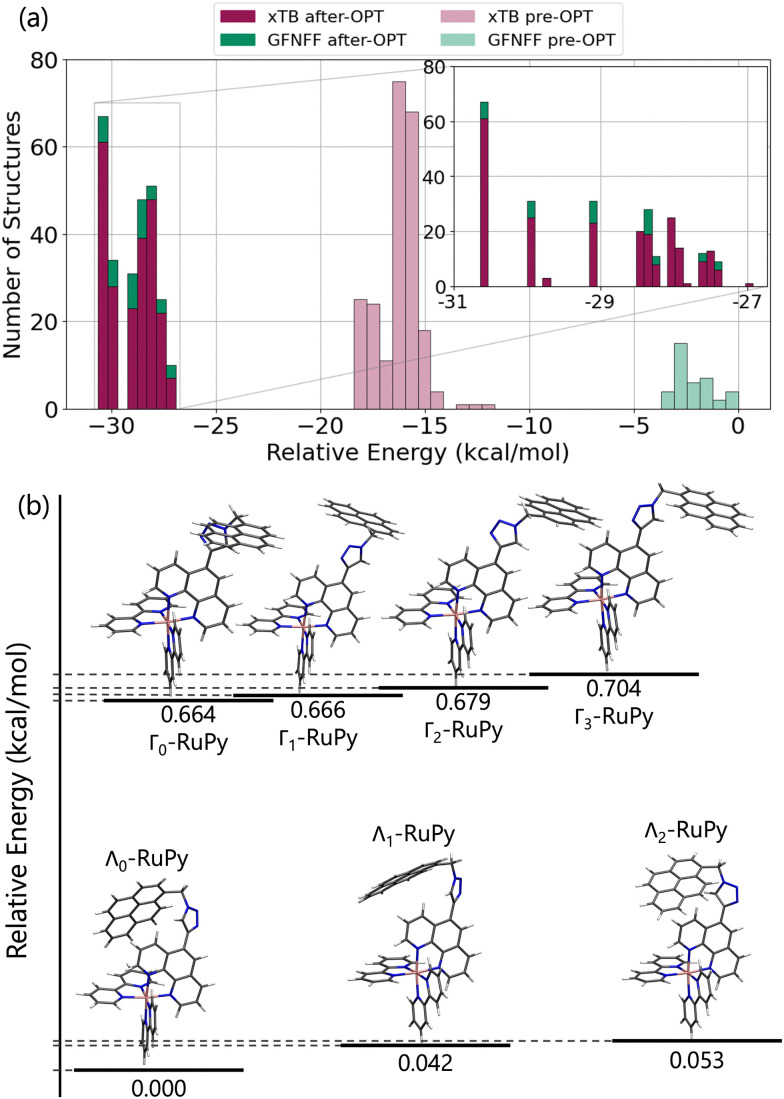
(a) Histogram of relative single-point energies in kcal mol^−1^ calculated at the B3LYP-D3(BJ)/def2-SVP of theory in implicit water for RuPy geometries generated with CREST. In light pink and light green, the relative energy of the structures obtained with CREST at the xTB and GFNFF levels of theory, respectively. In dark pink and dark green, the relative energies after re-optimization at the B3LYP-D3(BJ)/def2-SVP level of theory. A zoom-up of the most stable re-optimized structures is also displayed. (b) Relative total energies in kcal mol^−1^ and geometries representation of the seven most stable clusters’ representatives after CREGEN clustering algorithm. The reference corresponding to the lowest energy conformer is a Λ_0_-type of structure and has a total energy of −2550.67655 Hartree. The next two most stable – mostly degenerated – conformers are also Λ-shaped, followed by almost degenerated four Γ-shaped conformations.

First, we set out to find the possible conformations of RuPy in a water environment. Using CREST we identified 228 and 38 different conformers at the xTB and GFNFF levels of theory, respectively. The single point energy DFT calculations show that the structures obtained at the xTB level of theory are more stable energetically than those generated with GFNFF. Yet, regardless of the level of theory, all 266 structures converged to the same energy range after proper re-optimization at the DFT level of theory, as seen in Fig. 6a.

The CREGEN routine identified and removed 224 duplicate conformers from rotational constants and Cartesian RMSD, resulting in 42 unique conformers for further analysis, which were grouped into 39 different clusters. The 42 conformers are distributed within less than 4 kcal mol^−1^ above the lowest energy reference, which evidences the floppiness of the system. The lowest energy structures are displayed in Fig. 6b. The three most stable geometries were of Λ_*i*_-type. They evidence a strong interaction between the pyrene unit and the phenanthroline (phen) ligand, resulting in a closed geometry characterized by angle *θ* values smaller than 80. Despite the pyrene unit is known to intercalate when interacting with double stranded DNA structure,^26,28^ this closed geometry prevents the pyrene unit from intercalation. For this reason, only Λ_0_-RuPy was considered for further interaction studies, with the complex initially placed at external, major and minor groove binding positions.

The next four most stable structures are of Γ_*i*_-type and placed 0.7 kcal mol^−1^ above the Λ_0_-RuPy structures, recall Fig. 6b. These structures are characterized by *θ* values around 90. Accordingly, their open geometry is suitable for intercalation and thus all four geometries were further considered for the interaction studies. The *θ* angle and the three torsional angle values for each of the five selected structures are collected in Table 1.

**Table 1:** Angles *θ*, *ϕ*, *ψ* and *ω* (in degrees) of RuPy geometries (recall Fig. 1) selected for binding with DNA

	*θ* (°)	*ϕ* (°)	*ψ* (°)	*ω* (°)
Λ_0_-RuPy	58.6	−49.6	−31.3	−132.6
Γ_0_-RuPy	91.3	−66.0	−48.6	−30.5
Γ_1_-RuPy	90.6	65.7	47.2	29.0
Γ_2_-RuPy	90.0	65.4	45.0	−29.2
Γ_3_-RuPy	89.7	−65.2	−45.3	30.8

### 3.2. Binding of ruthenium(ii) complexes to DNA

#### 3.2.1. Initial trajectories from Λ_0_-RuPy and Γ_0_-RuPy structures

**Fig. 7:**
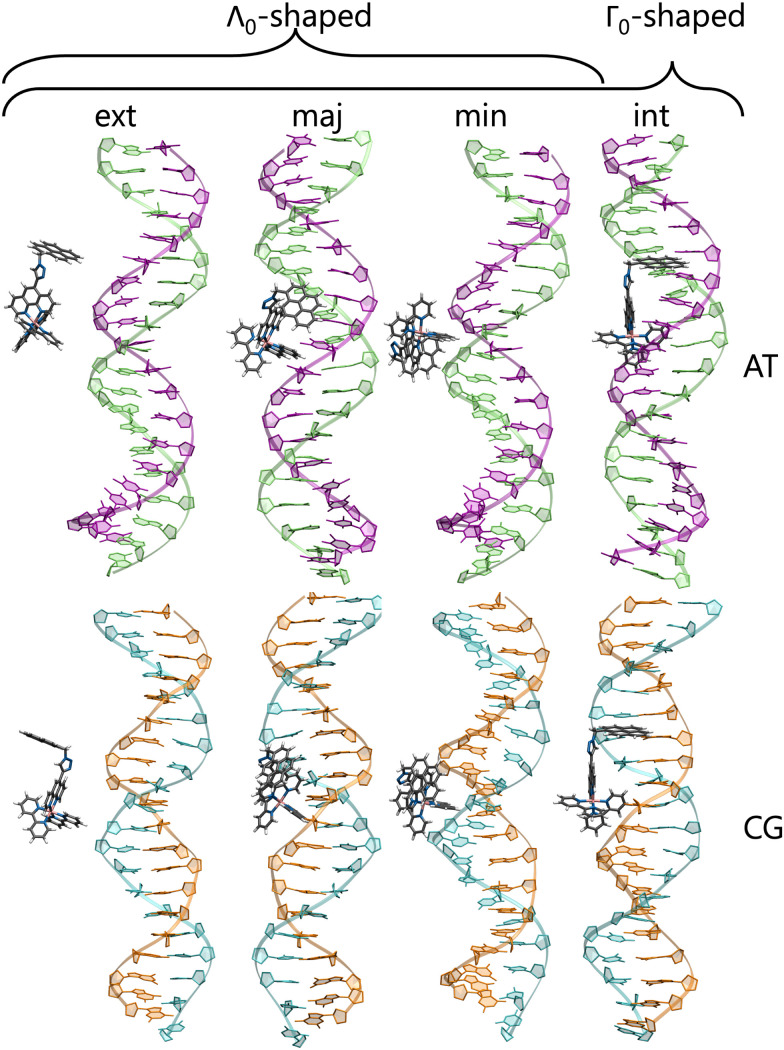
Initial structures of RuPy/DNA adducts. Λ_0_-RuPy conformations were initially bound externally as well as in the major and minor grooves binding of AT and CG sequences (6 trajectories, 3 for each DNA sequence). Γ_0_-RuPy structures are additionally intercalated (8 trajectories, 4 for each DNA sequence).

As indicated before, the Λ_0_-RuPy conformation was initially bound to AT and CG DNA double strands in the major and minor groove pockets, and externally, near the negatively charged phosphate backbone. Intercalation was not possible due to its closed conformation. Similarly, the Γ_0_-RuPy complex was attached externally, in the pocket grooves (minor and major) but also intercalated between four nucleobases from the major groove pocket. A scheme summarizing the initial conditions is shown in Fig. 7.

Henceforth, “convergence” or “steady state” will refer to the stability of the RuPy/DNA adduct, specifically when RuPy interacts with DNA in a consistent manner throughout the simulations. This is different from the various relative orientations RuPy can assume with respect to DNA, even when the interaction remains stable and involves the same atoms in the same binding mode, such as intercalation of the pyrene moiety between consecutive nucleobase pairs. Due to its three dihedral angles, RuPy can adopt multiple conformations, while DNA, as a macromolecule, possesses hundreds of degrees of freedom. Although it is expected that these conformations will vary from one time step to the next, the interaction between the metal complex and the DNA remains stable, thereby leading to adduct stability.

**Fig. 8:**
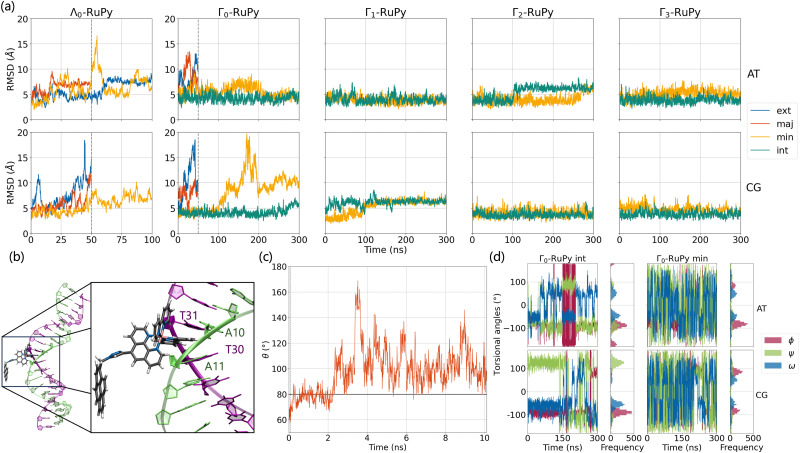
(a) RMSD values of production run trajectories computed against the starting structures for Λ_0_-RuPy and Γ_*i*_-RuPy with *i* = 0,1,2,3 structures initially placed interacting externally (ext), in the major groove pocket (maj), in the minor groove pocket (min) or intercalated between two nucleobase pairs (int) of both poly(dA)poly(dT) (top) and poly(dC)poly(dG) (bottom) DNA double strands. (b) Partial intercalation of a bpy ligand of Γ_0_-RuPy between the nucleobase pairs A10-T31 and A11-T30. (c) Time evolution of angle *θ* in the heating and equilibration stages of the simulation when Λ_0_-RuPy is initially placed in the major groove pocket of poly(dA)poly(dT) DNA double strand. The horizontal line at 80 indicates the threshold for distinguishing between Λ-shaped geometry (*θ* < 80) and Γ-shaped geometry (*θ* > 80). (d) Time evolution and histogram of *ϕ*, *ψ* and *ω* torsional angles along the production runs for Γ_0_-RuPy initially placed in intercalated between two nucleobase pairs (int) and in the minor groove pocket (min) of both poly(dA)poly(dT) (top) and poly(dC)poly(dG) (bottom) DNA double strands.

Fig. 8a shows the RMSD of DNA and the RuPy heavy atoms calculated against the starting structure. As it can be seen, most simulations starting with the Λ_0_-RuPy geometry did not reach a steady state in the external or groove binding modes considered. To further examine the relative position of the Λ_0_-RuPy complex with respect to the DNA, the number of short and long range contacts *N*_SRC_ and *N*_LRC_ were plotted along the equilibration (10 ns) and production (50 ns) stages of each simulation (Fig. S1 and S2 in the ESI†). In periods during which both *N*_SRC_ and *N*_LRC_ were zero, RuPy evolved to the bulk solvent. Intervals with *N*_SRC_ values below 150 and *N*_LRC_ non-zero were associated to RuPy interacting with the oligonucleotide externally, while higher values of *N*_SRC_ indicated that RuPy was in one of the groove pockets. *N*_SRC_ and *N*_LRC_ are linked to van der Waals and electrostatic interactions between RuPy and the DNA, respectively. From the number of contacts it follows that most initial set ups lead to intermittent interaction of RuPy with the DNA sequence. RuPy quickly changed to external binding mode and went to the bulk solvent, sometimes returning to external interaction with a different relative orientation. Likewise, the simulations with Γ_0_-RuPy geometries initially placed in external or major groove binding modes diverge (RMSD values above 7.5 Å). By contrast, the Λ_0_-RuPy structures initially interacting externally with the AT DNA double strand or bound in the major groove pocket exhibited small RMSD values (blue and red lines in Fig. 8a for Λ_0_-RuPy/AT- top left), even if the RuPy does not stay in its initial position. Indeed, RuPy initially placed in the major groove evolves to the end of the double strand where the bpy ligand can interact with A20-T21 base pairs by π–π stacking. This behaviour is an artificial binding due to the limited length of the DNA molecule; therefore, such trajectory was not considered for further analysis. Likewise, the trajectory that started from Λ_0_-RuPy in external binding mode with the AT DNA double strand also turned unstable. With RMSD around 7.5 Å during the first 50 ns, the trajectory was extended to 100 ns, but RuPy moved to the end of the AT double strand with the pyrene moiety held by π–π stacking interactions with the A1-T40 base pair; so this trajectory was also discarded.

The initially intercalated Γ_0_-RuPy/AT and Γ_0_-RuPy/CG adducts (green lines in Fig. 8a) and Γ_0_-RuPy/CG with RuPy bound to the minor groove pocket (yellow lines) equilibrated and reached a steady state along the first 50 ns of the production run. Despite larger RMSD values for AT than CG—mainly due to partial intercalation of the bpy ligand between the nucleobase pairs A10-T31 and A11-T30, see Fig. 8b—, the interaction of RuPy with the minor groove is stable. To confirm this change of binding mode and other potential ones, trajectories with Γ_0_-RuPy in minor groove and intercalative binding were extended up to 300 ns, thus providing better statistics. Intercalated metal complex in both DNA strands proved to form stable adducts. Larger RMSD values were found for RuPy bound to the minor groove pocket. As mentioned, for Γ_0_-RuPy/AT adduct this was due to partial intercalation of the bpy ligand which returned to the minor groove pocket after 200 ns. Γ_0_-RuPy/CG adduct was stable up to 100 ns, then evolving into the bulk and behaving as the complexes initially placed in the major groove or in external binding revealing minor groove binding not being as stable as intercalation. The results for the stable 300 ns extended trajectories will be discussed below in more detail.

Fig. 8c shows the evolution of angle *θ* along the heating and equilibration stages of the simulation for Λ_0_-RuPy/AT initially placed at the major groove. The initial *θ* value around 60 quickly evolves to 70 and then opens up beyond 80, which is the threshold to pass from Λ-shape to Γ-shape. By opening, RuPy evolves so that the pyrene interaction with the closest coordination sphere of RuPy (the ruthenium atom, the bpy ligands and the phen moiety) is not favoured anymore. This change of the *θ* angle was observed for all trajectories starting with Λ_0_-RuPy (Fig. S3 and S8 in the ESI†). Because of this behavior, Λ_1_ and Λ_2_-shaped geometries were not considered for interaction with DNA double strands as this shape was expected to change when interacting with the oligonucleotide, as seen for Λ_0_. By contrast, the angle *θ* for Γ_0_-RuPy/DNA trajectories remains above 90 (Fig. S4 to S7 and Fig. S9 to S12 in the ESI†). We thus can conclude that Λ-shaped geometries are not favoured to interact with DNA; they will be disregarded henceforth. This finding is not surprising, as the bending behavior is driven by the hydrophobic nature of the pyrene unit: due to its flexibility, the pyrene moiety tends to interact with the bpy ligands or the phen moiety of RuPy in a protic solution, such as water. By contrast, bound to DNA, the pyrene moiety finds an alternative, more energetically favorable way to minimize its contact with the protic solvent such that its interaction with the remainder of the RuPy complex ceases loosing the closed Λ shape.

Next, we analyze *ϕ*, *ψ* and *ω* torsional angles throughout all MD simulation stages for all trajectories where Γ_0_-RuPy was the initial RuPy geometry. This analysis aimed to determine whether the remaining three Γ_*i*_-RuPy geometries, with *i* = 1,2,3, are worth further investigating in order to achieve an accurate binding mode analysis. Fig. 8d shows the time-evolution and the distribution of the three torsional angles along the production run of the Γ_0_-RuPy complex, starting from the minor groove and intercalated in both AT and CG sequences. A significant difference in the distributions of the torsional angles was observed depending on the initial position of Γ_0_-RuPy. When Γ_0_-RuPy is intercalated (Fig. 8d left), *ϕ* is mostly distributed around-90 during the whole simulation, indicating its low probability to rotate to positive values for both AT and CG sequences. The angle *ψ* is centered around a negative value for AT, although there are signs that it can change to positive values even if this is not the preferred geometry. When interacting with CG, despite *ψ* being initially negative, it shifts to positive values, being this the most visited conformation. The torsional angle *ω* is primarily distributed around +50 or −50 indicating certain freedom of motion. From these results, we conclude that the Γ_0_ conformation interacts favorably with DNA and it is worth to be investigated in detail in the intercalative mode. Nonetheless, since the *ϕ* torsional angle remains mostly around its initial value, additional simulations should be performed with the remaining Γ_*i*_-RuPy geometries, with *i* = 1,2,3. Otherwise, we might disregard more favorable intercalative binding modes between different geometries of RuPy and DNA that are not energetically accessible when starting with the Γ_0_-RuPy geometry.

When initially placed in the minor groove pockets (Fig. 8d right), the three torsional angles freely explored the conformational space holding positive and negative values. This trend can easily be explained from the freedom of Γ_0_-RuPy in this particular binding mode regardless of the DNA sequence. Visualization of trajectories indicated that the interaction with the minor groove pocket was held by different parts of the Γ_0_-RuPy complex at different times of the simulation, *i.e.* the pyrene or triazole moieties, and the bpy or phen ligands. This allowed the torsional angles freedom of movement while maintaining a stable Γ_0_-RuPy/DNA adduct as interaction was always present.

Even though the freedom of the torsional angles in this binding mode covered the conformational space of RuPy, the behavior observed in the Γ_0_-RuPy/AT trajectory, which ultimately led to the intercalation of the bpy ligand, encouraged additional MD simulations to be performed aiming for better statistical data. These additional simulations where conducted for the remaining Γ_*i*_-RuPy geometries, with *i* = 1,2,3, as initial structures initially placed in the minor groove pocket of both AT and CG sequences.

To summarize, our simulations indicate that the Λ-shaped geometry is not relevant to form RuPy/DNA adducts, while the Γ_0_-shaped geometry becomes a stable adduct with DNA only in intercalative binding mode. Minor groove binding exhibited two distinct behaviours for the different DNA sequences: with AT the bpy ligand showed partial intercalation, while for the CG oligonucleotide RuPy left the minor groove exploring external and major groove binding modes as well. Further discussion on the Γ_0_-RuPy/DNA non-converged trajectories for external and major groove is provided in Section S4 of the ESI.† In the intercalative mode, the torsional angle *ϕ* did not vary significantly, indicating the need for additional simulations using the remaining Γ_*i*_-RuPy geometries (*i* = 1,2,3) as initial RuPy structures. In the minor groove binding mode, although non stability of the Γ_0_-RuPy/CG adduct was observed and the torsional angles showed considerable freedom of movement, further simulations with Γ_*i*_ geometries were additionally performed to improve statistical accuracy and because of the interesting behavior seen in the Γ_0_-RuPy/AT trajectory, where the bpy ligand exhibited partial intercalation between two nucelobase pairs. Table 2 summarizes all starting RuPy/DNA conditions and the stability of the resulting adduct in a particular binding mode scenario.

**Table 2:** Initial set-ups of RuPy/DNA adducts and their stability

DNA sequence				
RuPy	poly(dA)poly(dT)		poly(dC)poly(dG)	
	Binding mode	Converged	Binding mode	Converged
Λ_0_	External	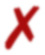	External	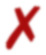
	Major groove	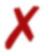	Major groove	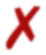
	Minor groove	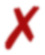	Minor groove	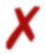
Γ_0_	External	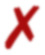	External	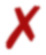
	Major groove	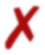	Major groove	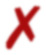
	Minor groove	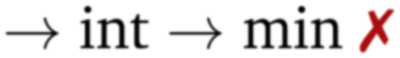	Minor groove	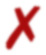
	Intercalation	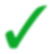	Intercalation	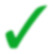
Γ_1_	Minor groove	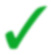	Minor groove	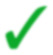
	Intercalation	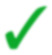	Intercalation	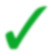
Γ_2_	Minor groove	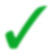	Minor groove	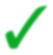
	Intercalation	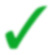	Intercalation	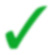
Γ_3_	Minor groove	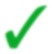	Minor groove	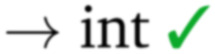
	Intercalation	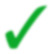	Intercalation	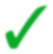

#### 3.2.2. Intercalative and minor groove binding modes

Here we further discuss the trajectories corresponding to the four Γ_*i*_-RuPy geometries, which were intercalated and bounded into the minor groove of the DNA. The 16 different RuPy/DNA adducts were equilibrated during 10 ns, followed by 300 ns production runs, proving to be steady adducts (Fig. 8a), with the exception of Γ_0_-RuPy/CG initially in the minor groove. For each stable trajectory, binding free energies (Tables 3 and 4) were calculated within MM-PBSA approach using both STP and 3TP. As one can see, the general trend is that intercalation yields more stable binding free energies than minor groove binding, despite some values obtained with the 3TP highly deviate from the norm-behaviour that will be later discussed in more detail.

**Table 3:** Binding free energies in kcal mol^−1^ for all four Γ-RuPy geometries initially placed in the minor groove pocket or intercalated between nucleobase pairs of poly(dA)poly(dT) DNA double strands calculated with the MM-PBSA approach with the single trajectory protocol (STP) or the three trajectory protocol (3TP) and the standard errors (SE)

Initial geometry	Int				Min			
MM-PBSA protocol	STP	SE	3TP	SE	STP	SE	3TP	SE
Γ_0_-RuPy	−24.91	0.06	−6.97	0.75	−13.8^a^, −21.25^b^	0.23^a^, 0.08^b^	−8.40^a^, −7.75^b^	1.58^a^, 0.86^b^
Γ_1_-RuPy	−21.94	0.06	−18.40	0.75	−8.05	0.06	−6.64	0.76
Γ_2_-RuPy	−18.61	0.06	−15.77	0.74	−8.49	0.08	−7.14	0.75
Γ_3_-RuPy	−20.62	0.08	−1.88	0.77	−18.59	0.12	−5.64	1.00

a Only relative geometries of the RuPy/DNA adducts with minor groove binding approaching through the minor groove considered.

b Only relative geometries of the RuPy/DNA adducts with intercalative binding approaching through the minor groove considered.

**Table 4:** Binding free energies in kcal mol^−1^ for all four Γ-RuPy geometries initially placed in the minor groove pocket or intercalated between nucleobase pairs of poly(dC)poly(dG) DNA double strands calculated with the MM-PBSA approach with the single trajectory protocol (STP) or the three trajectory protocol (3TP) and the standard errors (SE)

Initial geometry	Int				Min			
MM-PBSA protocol	STP	SE	3TP	SE	STP	SE	3TP	SE
Γ_0_-RuPy	−21.20	0.07	−17.37	0.76	—	—	—	—
Γ_1_-RuPy	−20.12	0.06	−13.93	0.75	−10.16	0.07	−7.08	0.78
Γ_2_-RuPy	−21.31	0.07	−16.37	0.76	−14.76	0.06	−13.01	0.76
Γ_3_-RuPy	−22.08	0.07	−18.18	0.76	−11.4^a^, −26.22^b^	0.12^a^, 0.08^b^	−7.00^a^, −20.73^b^	1.44^a^, 0.90^b^

a Only relative geometries of the RuPy/DNA adducts with minor groove binding approaching through the minor groove considered.

b Only relative geometries of the RuPy/DNA adducts with intercalative binding approaching through the minor groove considered.

##### Intercalative binding

**Fig. 9:**
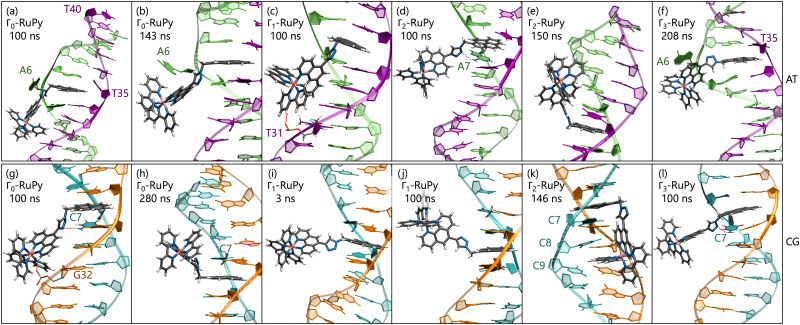
Snapshots of the MD simulations with all Γ_*i*_-RuPy with *i* = 0,1,2,3 initially intercalated in both poly(dA)poly(dT) (a–f) and poly(dC)poly(dG) (g–l) DNA double strands. Color code for atoms in RuPy and residues in DNA: grey for C, blue for N, pink for Ru, white for H, green for adenine, purple for thymine, light blue for cytosine, and orange for guanine.

All simulations with RuPy initially intercalated led to stable RuPy/DNA adducts (see green RMSD values in Fig. 8a). The strong interaction between the RuPy complex and the oligonucleotide can be attributed to π–π stacking between the pyrene moiety and the two intercalative nucleobase pairs. Additional stability along some parts of the production runs comes from van der Waals interactions with the major groove and electrostatic interactions with the negatively charged phosphate backbone although not being strong enough to provide stability throughout the whole simulation. In the following, we discussed the different conformations adopted by all Γ-RuPy/DNA adducts throughout the MD trajectories. Fig. 9 depicts representative configurations of these trajectories. Naturally, as these structures are captured at an specific instant in time, they will change slightly upon further propagation. Nevertheless, they serve as useful representations of most common configurations of the adducts.

The adducts consisting of Γ_0_-RuPy and Γ_3_-RuPy with AT experienced a rupture of the Watson–Crick pairing of the A6-T35 base-pair adjacent to the intercalation site, allowing the pyrene moiety of RuPy for better intercalation between the two neighbouring nucleobase-pairs and interact by π–π stacking with them. This behaviour was stabilized by the formation of stacking interactions between the replaced A6 nucleobase and the triazole moiety (Fig. 9a), a bpy ligand (Fig. 9b) or the phen ligand (Fig. 9f). However, no interactions were observed between the pyrene moiety and any T nucleobase such that the A-T pairing was shifted by one T nucleobase resulting in non-paired T40 at the end of the DNA double strand (Fig. 9a).

In the Γ_1_-RuPy/AT adduct (Fig. 9c), the [Ru(bpy)_2_]^2 +^ moiety oriented along the DNA backbone, providing extra stability due to intermittent interactions between the bpy ligands of RuPy and the oxygen atoms in the DNA phosphate backbone, and electrostatic interactions between the positively charged RuPy and the negatively charged DNA phosphate backbone.

RuPy in the Γ_2_-RuPy/AT adduct during the first 100 ns kept the original geometry in which it was initially placed. An hydrogen bond between a nitrogen atom in the triazole moiety of RuPy and a hydrogen atom in the A7 gave further stabilization in some stages of the trajectory (Fig. 9d). In others, hydrogen atoms of one of the bpy ligands interacted with the oxigen atoms of the phosphate backbone. Intermittent interaction of the bpy ligands with the DNA backbone was also observed combined with van der Waals interaction with the major groove. After 100 ns, the torsional angle *ϕ* changed its value observed as a flip on the relative orientation with respect to the DNA axis (see Fig. 9e and Fig. S16 in the ESI†) which can also be noted in a higher but stable value of RMSD, as it was computed against the starting structure.

Regarding the CG sequence, no special features were observed for Γ_3_-RuPy/CG adduct (Fig. 9l). RuPy was stable at the position initially intercalated and an hydrogen bond between a nitrogen atoms in RuPy triazole moiety and an hydrogen atom in C7 was observed in the simulation, giving extra stability to the adduct. These interactions were also noted for the vast majority of Γ_0_-RuPy/CG and Γ_2_-RuPy/CG adducts trajectories (Fig. 9g). The Γ_2_-RuPy/CG adduct proved that dual intercalation of the pyrene moiety and a bpy ligand is not stable. The bpy ligand exhibited partial intercalation by interacting with a cytosine nucleobase (C8) two steps away from the pyrene intercalation site (see Fig. 9k). However, this interaction disappears after several nanoseconds. The instability is likely due to the energetic penalty associated with disrupting the DNA double helix, as partial intercalation can cause base-pair unstacking, which is energetically unfavorable.^64^ Consequently, the interaction between the bpy ligand and the nucleobase was not maintained, which agrees with bpy being known as partial intercalator or metallo-groove binder.^65,66^ The trajectory of Γ_1_-RuPy/CG adduct showed weaker interaction of the closest coordination sphere of RuPy with the CG double strand (Fig. 9i), allowing for *ϕ* torsional angle to change its value observed as a flip on the relative orientation with respect to the DNA axis (see Fig. 9j and Fig. S20 in the ESI†) which is linked to a higher RMSD value. This behaviour was also observed for the Γ_0_-RuPy/CG adduct by the end of the simulation (around 280 ns, see Fig. 9h.) None of the CG simulations exhibit rupture of Watson–Crick pairing, which agrees to the relative strength of AT and CG pairs.

The angle *θ*, initially around 90 in all cases, shifts to higher values for all RuPy/DNA adducts, ranging from 80 up to 175 (Fig. S4–S7 and Fig. S9–S12 in the ESI†). For small *θ* angles, the RuPy complex excluding the pyrene moiety—the ruthenium atom with its attached bpy and phen ligands, and the triazole—interacts strongly with the DNA major groove (see the case of Γ_1_-RuPy/AT in Fig. S5 in the ESI†). Values of *θ* being around 175 correspond to adducts where the interaction of the portion of the RuPy complex excluding the pyrene moiety with DNA is not as favoured (as in the case of Γ_1_-RuPy/CG adduct in Fig. S10 in the ESI†). The torsional angle *ϕ* keeps its starting value for almost all intercalative Γ_*i*_-RuPy/DNA adducts, with the exception of Γ_2_-RuPy/AT, Γ_0_-RuPy/CG and Γ_1_-RuPy/CG, as already mentioned. In this case, as mentioned above, the complex unbends due to weak interaction with the CG double strand, giving the torsional angle *ϕ* freedom to flip. Thus, it could be concluded that although *ϕ* is most likely to remain fixed it can change its value if interactions between RuPy and the DNA double strand are not strong enough due to *e.g.* breathing of the DNA double strand. The torsional angles *ψ* and *ω* are more flexible, but it is more difficult to draw correlations between their starting and final values.

All binding free energies obtained within the MM-PBSA approach with the STP are in the range of −18.61 to −24.91 kcal mol^−1^ for AT, both with standard error (SE) of 0.06, (Table 3). For the CG sequence, they range from −20.12 to −22.80 kcal mol^−1^, with an SE of 0.06 (Table 4). Lower binding energies are related to trajectories were the flip of torsional angle *ϕ* was observed. Since the average binding free energies for AT (−21.5 ± 2.3 kcal mol^−1^) and CG (−21.18 ± 0.70 kcal mol^−1^) are very similar, no significant difference in relative stability between the two nucleotides can be inferred, although the larger variability in the AT sequence suggests more fluctuations in those adducts. In contrast, when the 3TP was employed, binding free energies ranged from −1.88 to −18.40 kcal mol^−1^ for AT, with SE values of 0.77 and 0.75, and from −13.93 to −18.18 kcal mol^−1^ for CG, with SE 0.75 and 0.76. The wider range in the 3TP results is due to accounting for the conformational changes in both RuPy and the DNA strands, which are not captured in the STP. While considering that these conformational changes can improve the accuracy of binding free energy estimates, there are cases where the resulting values appear unrealistic. For instance, the binding free energies for Γ_0_-RuPy/AT and Γ_3_-RuPy/AT are −6.97 and −1.88 kcal mol^−1^, respectively, with SE values of 0.85 and 0.77. In these cases, the AT double strand experienced a breakage in the Watson–Crick pairing, which was considered in the adduct trajectory but not in the isolated AT simulation. Notably, this breakage is not a mandatory condition for RuPy intercalation but a consequence of these specific simulations. Considering these observations, while the 3TP theoretically provides a better approximation to true binding free energies, it is not always more reliable in practice, and inspecting each individual case is beneficial. In general, the predicted binding free energies obtained using 3TP are larger than those obtained with STP, reflecting the fact that upon association, the RuPy complex and DNA adapt to each other. This adaptation results in a more favorable energetic state in the bound form compared to their respective unbound conformations, which are sampled separately in the 3TP. Consequently, the free energy of conformations extracted from separate trajectories (representing the unbound states) is expected to be higher than that of conformations from the bound complex trajectory (representing the bound state).

##### Minor groove binding

Simulations with RuPy placed in the minor groove pocket also yielded stable adducts (see low RMSD values in Fig. 8a). An exception was observed for Γ_0_-RuPy/CG, which drifted into the bulk solution, intermittently interacting with the major groove before ultimately binding to the terminal nucleobase pair of the DNA.

Angle *θ* exhibited greater variations than in the intercalative adducts (Fig. S4–S7 and Fig. S9–S12 in the ESI†), due to the nature of the interactions between RuPy and DNA. In intercalation, the pyrene moiety directly interacts with DNA, whereas in the minor groove, the stability of the adduct can be attributed to interactions involving the bpy ligands, the pyrene moiety, or both with the oligonucleotide. This range of potential interactions provides more flexibility to the complex, resulting in larger *θ* values. The same applies to the torsional angles *ϕ*, *ψ*, and *ω*, which explore a larger conformational space.

**Fig. 10:**
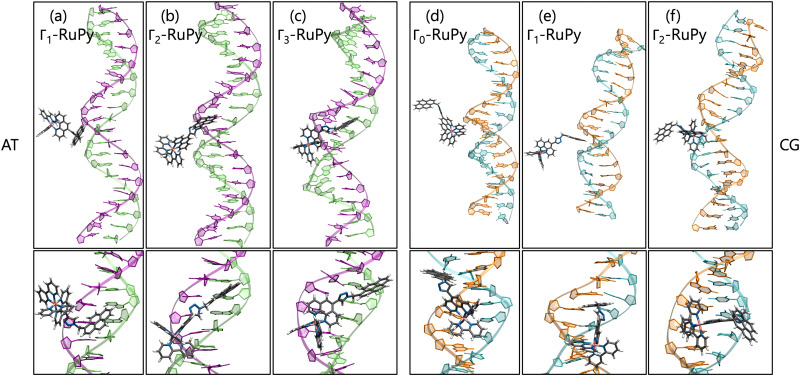
Snapshots at time 100 ns in two different perspectives of the MD simulations with all Γ_*i*_-RuPy with *i* = 0,1,2,3 initially placed in the minor groove pocket of both poly(dA)poly(dT) (a–c) and poly(dC)poly(dG) (d–f) DNA double strands. Trajectories that converged to intercalative binding mode are not shown. Color code for atoms in RuPy and residues in DNA: grey for C, blue for N, pink for Ru, white for H, green for adenine, purple for thymine, light blue for cytosine, and orange for guanine.

Different orientations of RuPy with respect to DNA were observed throughout the trajectories of all adducts (Fig. 10), despite all Γ_*i*_-RuPy structures starting in the same relative orientation—with the pyrene moiety of RuPy facing the DNA minor groove (recall Fig. 3). At various stages, the interaction between RuPy and the minor groove was established through the pyrene moiety, which adopted orientations either perpendicular to the DNA backbone (Fig. 10a) or perpendicular to the DNA axis (Fig. 10e). In addition to the pyrene moiety, the triazole moiety also formed strong interactions with the minor groove, particularly in the Γ_2_-RuPy/AT adduct (Fig. 10b). Meanwhile, the bpy ligands interacted significantly with the minor groove in the Γ_3_-RuPy/AT adduct, leading to substantial deformation of the groove (Fig. 10c). In the Γ_0_-RuPy/CG and Γ_2_-RuPy/CG adducts (Fig. 10d and f), strong interactions were observed between the bpy ligands of RuPy and the minor groove, while the pyrene moiety remained oriented outward from the DNA, exhibiting freedom of movement due to its lack of interaction. In the case of Γ_0_-RuPy/CG, however, the interaction was weaker, causing RuPy to drift into the bulk solvent after 100 ns.

The behavior observed for minor groove binding is consistent with the binding free energies obtained using the MM-PBSA methodology, which are generally higher than those observed for the intercalative binding mode. This suggests that the interaction of RuPy with the minor groove is weaker compared to the stronger π–π interactions between the pyrene moiety and the nucleobase pairs, also evidenced by the Γ_0_-RuPy/CG trajectory evolving to the bulk solvent. Specifically, binding free energies for the minor groove binding range from −5.64 to −14.76 kcal mol^−1^ (considering both STP and MTP) with standard errors of 1.00 and 0.06. An exception is the Γ_3_-RuPy/AT binding free energy obtained *via* STP, which measured −18.59 kcal mol^−1^ with a standard error of 0.12. In most trajectories, deformation of the minor groove was minimal, resulting in similar binding free energies for both protocols. However, the Γ_3_-RuPy/AT adduct showed a marked difference between STP and MTP binding free energies. In this case, RuPy maintained its orientation relative to the oligonucleotide, leading to a lower binding free energy in the STP approach. In contrast, the conformational change in the minor groove, necessary to accommodate the complex, resulted in a higher binding free energy in the MTP approach.

The resulting average binding free energies obtained with the STP were −10.1 ± 2.6 kcal mol^−1^ for the AT sequence and −12.1 ± 1.9 kcal mol^−1^ for the CG sequence (excluding the lower value for Γ_3_-RuPy/AT). For the 3TP approach, the averages were −6.96 ± 0.99 kcal mol^−1^ for AT and −9.0 ± 2.8 kcal mol^−1^ for CG. Similar to the intercalative binding case, there is no clear preference for either AT or CG sequences. Therefore, the variations in binding free energies observed in the MM-PBSA STP approach cannot be linked to a specific DNA sequence, but rather to the distinct manner in which RuPy interacts with the minor groove pocket. As expected, the binding free energies calculated using the 3TP method are generally higher due to the inclusion of conformational changes in the system.

**Fig. 11:**
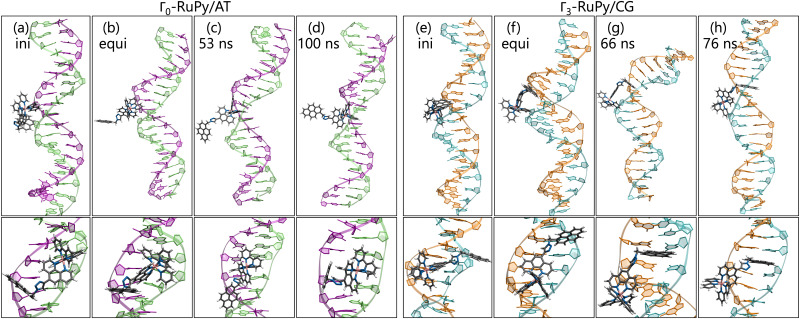
Snapshots in two different perspectives of the MD simulation trajectory where (a)–(d) Γ_0_-RuPy was initially placed at poly(dA)poly(dT) minor groove pocket but evolved to intercalation of the bpy ligand and (e)–(h) Γ_3_-RuPy initially placed at poly(dC)poly(dG) minor groove pocket ends up intercalating by the pyrene moiety ligand. Color code for atoms in RuPy and residues in DNA: grey for C, blue for N, pink for Ru, white for H, green for adenine, purple for thymine, light blue for cytosine, and orange for guanine.

Distinct behavior was observed for the Γ_0_-RuPy/AT adduct (Fig. 11a–d). Although RuPy was initially placed with the pyrene moiety facing the DNA (Fig. 11a), it explored the entire phase space during the equilibration stage. Strong interactions were established between RuPy's closest coordination sphere and the minor groove pocket (Fig. 11b), stabilized by van der Waals forces with the bpy ligands and electrostatic interactions with RuPy's positive charge. After 50 ns of the production run, partial intercalation of a bpy ligand was observed between the nucleobase pairs A10-T31 and A11-T30 (Fig. 11c), which was stabilized by π–π stacking interactions (Fig. 11d). However, shortly after 200 ns, the intercalation of the bpy ligand was no longer favored, and RuPy returned to minor groove binding. This behavior can be attributed to the nature of the bpy ligand, which acts as either a partial intercalator or a minor groove binder due to its lack of an extended conjugated aromatic system.^65,66^

Similarly, Γ_3_-RuPy/CG also exhibited special behavior (Fig. 11e–h). Initially, the pyrene moiety was oriented inward (Fig. 11e) and remained in the minor groove pocket, with its plane perpendicular to the nucleobase pairs (Fig. 11f). The “breathing” motion of the CG double strand facilitated the partial intercalation of the pyrene moiety between nucleobase pairs C7-G34 and C8-G33 (Fig. 11g), eventually leading to full intercalation. This final state was stabilized by π–π stacking interactions between the pyrene moiety and the nucleobase pairs (Fig. 11h).

Since the behaviour of these two trajectories corresponded to two different situations (RuPy placed in the minor groove and to RuPy intercalated, in Γ_0_-RuPy/AT by the bpy ligand and in Γ_3_-RuPy/CG by the pyrene moiety), the calculation of binding energies was correspondingly adapted.

Using the STP approach, the binding free energies for minor groove binding were −13.8 kcal mol^−1^ with SE 0.23 for Γ_0_-RuPy/AT and −11.4 kcal mol^−1^ with SE 0.12 for Γ_3_-RuPy/CG, while the values for intercalation were −21.25 and −26.22 kcal mol^−1^, respectively both with SE 0.08 (Tables 3 and 4). These results align with the binding free energies observed for both intercalation from the major groove and minor groove binding.

Binding free energies obtained *via* the 3TP approach for Γ_0_-RuPy/AT and Γ_3_-RuPy/CG were −8.40 and −7.00 kcal mol^−1^ with SE 1.58 and 1.44 for minor groove binding, and −7.75 and −20.73 kcal mol^−1^ with SE 0.86 and 0.90 for intercalation, respectively (Tables 3 and 4). The results follow the same trend as the STP, with the exception of Γ_0_-RuPy/AT during bpy ligand intercalation. The destabilization of the binding free energy can be attributed to the significant deformation required for the bpy ligand to intercalate between the nucleobase pairs, offering a more accurate estimate of the true binding free energy compared to STP. Unlike previous cases, where large increases in binding free energy were considered outliers, intercalation in this scenario would not be feasible without significant DNA deformation. On the other hand, the results for Γ_3_-RuPy/CG suggest that intercalation of the pyrene moiety from the minor groove might be more favorable than from the major groove. This is evidenced by consistently lower binding energies across both protocols when RuPy approached from the minor groove, suggesting a more energetically stable interaction in this orientation.

## 4 Conclusions

We have reported the binding of [Ru(2,2′-bipyridine)_2_ (5-{4-[(pyren-1-yl)methyl]-1*H*-1,2,3-triazol-4-yl}-1,10-phenanthroline)]^2+^ (RuPy), a complex featuring a flexible pyrene moiety, to DNA. We use classical molecular dynamics simulations to explore the different non-covalent binding interactions of RuPy with two different 20 base pair DNA sequences, poly(dA)poly(dT) (AT) and poly(dC)poly(dG) (CG).

RuPy conformers were initially positioned at various binding sites: externally interacting with the nucleotide, within the major and minor groove pockets, and with the pyrene moiety intercalated between two consecutive nucleobase pairs of DNA. Our results indicate that external interaction and major groove binding does not result in stable adducts for any of the DNA sequences, whereas intercalation consistently leads to stable complexes. Minor groove binding, however, presented more variability. While some trajectories yielded stable adducts, others were only semi-stable. In certain cases, RuPy transitioned from the minor groove pocket to intercalation, with either the pyrene unit or a bpy ligand being the intercalated unit. The binding free energies for RuPy in the minor groove prior to intercalation were comparable to those where RuPy remained in the groove, while intercalative binding involving the pyrene moiety was energetically similar to systems where intercalation occurred from the major groove. The results suggest that intercalation of the pyrene moiety from the minor groove is more favorable than from the major groove, indicating a more stable interaction in this orientation. In contrast, intercalation of the bpy ligand was not maintained and was associated with higher binding free energies, indicating that this mode of binding is less favorable than pyrene intercalation.

Relative binding free energies were calculated using the molecular mechanics Poisson–Boltzmann surface area (MM-PBSA) approach with both single trajectory (STP) and three trajectory (3TP) protocols. Intercalative binding consistently showed more favorable binding free energies compared to minor groove binding for both STP and 3TP. This trend is attributed to π–π interactions between the pyrene moiety and the DNA nucleobases, which stabilize the interaction. No significant difference could be inferred from the used of the AT and GC DNA sequences. When comparing the values obtained with the two protocols, 3TP generally yielded less negative binding free energies than STP. This reflects the conformational changes that both RuPy and DNA undergo upon interaction, indicating that binding free energies are often overestimated with the STP. However, the increase in energy was found to be unusually large in some cases where conformational changes were the result rather than the requirement for intercalative interaction, highlighting the need to inspect each case individually. The minor groove binding energies varied more widely for both STP and 3TP, reflecting the diverse interaction fashions between RuPy and the binding pocket. For sake of completeness, a mixed poly(dCATG) DNA double strand sequence has been considered in proof. The results, described in Section S7 (ESI†), show that RuPy complex remains effectively intercalated also in this case and minor group binding is less stable.

These findings contribute to the understanding of RuPy's binding modes to DNA, which can be further exploited for photocatalysis and as a versatile DNA probe. Experimental validation could provide additional insights into the applicability of such complexes in therapeutic and diagnostic settings. Future work will focus on the calculation of photophysical properties of the stable RuPy/DNA adducts to be applied as photosensitizers for photocatalysis.

## Author contributions

MM was in charge of performing all calculations, data curation, analysis, visualization, methodology and writing the original draft. LG conceptualized and supervised the work, discussed the results, edited the draft and acquired funding.

## Data availability

Relevant files and data underpinning this publication are openly available at https://github.com/merimp/RuPy-DNA.git. Other additional data including a description of files and data types have been included as part of the ESI.† The software codes to carry out the calculations are described in the Computational Details section.

## Conflicts of interest

There are no conflicts to declare.

## Supplementary Material

CP-026-D4CP02782E-s001
