# Supplementary material for: Binding modes of a flexible ruthenium polypyridyl complex to DNA
Source: Phys Chem Chem Phys. 2024 Oct 11;26(42):27116–30. doi: 10.1039/d4cp02782e (PMC11492816; doi:10.1039/d4cp02782e)
Supplement: CP-026-D4CP02782E-s001 [file CP-026-D4CP02782E-s001.pdf]

Supplementary Information (SI) for Physical Chemistry Chemical Physics.
This journal is © the Owner Societies 2024

SUPPORTING INFORMATION to: Binding Modes of a Flexible Ruthenium Polypyridyl Complex to DNA

Meritxell Malagarriga and Leticia González

October 1, 2024

Contents

S1 CREST **S2**

S2 MD simulations for Spontaneous Association **S2**

S3 Single and Multiple Trajectory Protocol for MM-PBSA Calculations **S2**

S4 External and Major Groove Binding **S4**

S5 Angle θ Evolution **S7**

S6 Torsional Angles Evolution **S12**

S7 RuPy/CATG **S18**

S1 CREST

The conformer-rotamer ensemble sampling of the molecule was performed using the CREGEN tool within the CREST software suite (version 3.0pre) at the GFN2-xTB level of theory. The input structure, consisting of 96 atoms, was processed, yielding 266 points in the XYZ files. A stringent RMSD threshold of 0.1250 Å and a conformer energy window of 6.0000 kcal/mol were applied to filter the conformers. The CREGEN routine identified and removed 224 duplicate conformers, resulting in 42 unique conformers for further analysis. The lowest energy conformer was set as the reference with a total energy of -2550.67655 Hartree. The relative energies of the conformers ranged up to 3.715 kcal/mol above the reference. The ensemble average energy was calculated to be 0.507 kcal/mol, with an ensemble entropy of 20.423 J/mol K and a free energy of -1.455 kcal/mol. The population of the lowest energy conformer was 20.161%.

S2 MD simulations for Spontaneous Association

To check for possible spontaneous associations an ensemble of non-interacting RuPy-DNA systems was set up. Eight different starting structures were considered - four for each DNA strand (AT and CG) - where Λ_0-RuPy geometry was placed such that its center of mass was at 20 Åfrom the DNA's center of mass and distributed around the DNA's axis in angles of 90 to cover for possible spontaneous minor and major groove binding, as well as intercalation. Λ_0-RuPy geometry was selected because, being the most stable in water and placing it at a reasonable distance from the DNA strand it would better resemble a supposed experimental case. The choice of just selecting four different angles was made with the aim to cover most of the DNA circle perimeter centered around the helical axis. The MD simulation protocol followed the same procedure outlined in Section 2.2, except for employing hydrogen mass repartition. This adjustment allowed us to increase the simulation time step from 2 fs to 4 fs, enabling the exploration of longer time scales without increasing computational cost. Specifically, we extended our production runs up beyond 1 μs and snapshots were recorded every 200 ps.

In seven out of the eight systems considered, RuPy ended up moving to the end of the DNA strand and interacting by π-π interactions with the terminal nucleobase pair. Although this behaviour is understandable given the strong and stable interaction that does not require the DNA to deform to accommodate the RuPy, it is nevertheless an artifact of the limited chain length using in the simulations. One of the trajectories yielded bpy intercalation approaching by the minor groove with a relative binding free energy of -23.68 kcal/mol and standard error 0.04, computed with the single trajectory protocol as no such long trajectories were run for isolated RuPy and DNA strands. However, we also know from our simulations on the Γ_0-RuPy/AT adduct that this type of interaction is not stable, with the bpy ligand leaving the intercalation site after several nanoseconds. Furthermore, the binding free energy calculated with the 3TP for this system confirmed the destabilization caused by the conformational changes required to accommodate intercalation, further highlighting the instability of this interaction mode. In agreement with other studies,[1] we concluded that the simulation times are not enough to simulate spontaneous intercalation of any ligand into the DNA. Given that in Ref. [1], they sampled > 93 μs and they were unable to capture intercalation at non-terminal base-pairs, owing to the simulations "not being long enough", we think that this approach is also not viable and manual docking approach remains both suitable and pragmatic in our case.

S3 Single and Multiple Trajectory Protocol for MM-PBSA Calculations

In our study, we employed both the single-trajectory protocol (STP) and the three-trajectory protocol (3TP) within the MM-PBSA approach to calculate the binding free energies of the

RuPy/DNA adducts. It is well acknowledged that the STP can sometimes overestimate binding energies due to the inherent limitations of using a single conformational ensemble, where conformational changes of the receptor and ligand upon binding are not fully accounted for. In contrast, the 3TP explicitly considers these conformational changes, potentially leading to more accurate predictions of binding free energies.

The predicted binding free energies obtained using the three-trajectory protocol (3TP) are generally larger than those obtained using the single-trajectory protocol (STP). This difference can be attributed to the inherent nature of the binding process. Upon association, the RuPy complex and the DNA adapt to each other, resulting in a conformational state that is energetically more favorable in the bound form than in their respective unbound forms. Consequently, the free energy of the conformations captured from separate trajectories (representing the unbound states) is expected to be lower than that of the conformations extracted from the complex trajectory (representing the bound state). This difference leads to a less favorable estimation of binding free energy in the 3TP case compared to the STP.

However, we have also investigated the influence of the stages of the trajectories considered for the MM-PBSA calculation. Our results, detailed in Table S1, show that the predicted binding free energies using the STP for the Γ_1-RuPy/DNA adducts were relatively stable across different stages of the first 200 ns of the production trajectory. For instance, the binding free energies for the AT sequence ranged from -23.50 to -21.19 kcal/mol, with a standard error (SE) consistently below 0.26. This stability is indicative of the STP's ability to yield consistent results due to the cancellation of errors in conformational energies, particularly when the RuPy complex remains in a stable binding mode such as intercalation. The electrostatic contributions and nonpolar solvation energies are well compensated in this method, leading to relatively small fluctuations in the predicted binding energies.

On the other hand, the 3TP results exhibit significant fluctuations in the predicted binding free energies, especially for the CG sequence, where values ranged from -22.11 to -9.48 kcal/mol. The difference of approximately 12.63 kcal/mol between the highest and lowest binding free energies reflects the impact of conformational sampling and the challenges associated with accurately capturing the adaptation energies of the RuPy complex and DNA. These fluctuations can be attributed to the difficulty in sampling enough conformations of the RuPy/DNA adducts in the low-energy conformational space, leading to less stable predictions of the adaptation energy when using the 3TP.[2]

In conclusion, our study demonstrates that both the STP and 3TP have distinct and complementary roles in analyzing the binding free energies of RuPy/DNA adducts. The STP provides consistent and stable predictions, making it valuable for assessing the overall stability of the binding mode across the trajectory. This consistency is particularly useful when the binding interaction remains stable, as seen in our simulations. However, the 3TP offers crucial insights into the dynamic conformational changes of both the RuPy complex and the DNA upon binding, capturing effects that the STP might overlook. These conformational changes can significantly impact the binding free energies, as reflected in the larger fluctuations observed with the 3TP.

Therefore, employing both protocols is essential for a comprehensive understanding of the binding interactions in flexible systems like RuPy/DNA adducts. The STP gives us a reliable baseline of the interaction stability, while the 3TP allows us to explore the conformational adaptability and its influence on binding energetics. Together, these approaches provide a more complete picture of the RuPy/DNA interaction landscape, ensuring that we account for both the stability of the binding modes and the flexibility of the interacting partners.

Table S1: Binding free energies in kcal/mol for Γ_1-RuPy geometry initially placed intercalated between nucleobase pairs of poly(dA)poly(dT) and poly(dC)poly(dG) DNA double strands calculated with the MM-PBSA approach with the single trajectory protocol (STP) or the three trajectory protocol (3TP) by taking different stages of the trajectory.

	**Γ_1-RuPy/AT int**				**Γ_1-RuPy/CG int**			
**Stage**	**STP**	**SE**	**3TP**	**SE**	**STP**	**SE**	**3TP**	**SE**
**0-20 ns**	-23.50	0.20	-19.10	2.84	-18.07	0.22	-14.39	2.77
**20-40 ns**	-22.38	0.20	-18.37	2.92	-18.10	0.19	-9.48	2.69
**40-60 ns**	-23.49	0.19	-20.82	2.75	-21.00	0.22	-15.40	2.81
**60-80 ns**	-22.47	0.21	-17.42	2.52	-20.13	0.19	-13.82	2.73
**80-100 ns**	-23.24	0.19	-15.35	2.70	-18.85	0.18	-11.06	2.49
**100-120 ns**	-22.86	0.26	-17.03	2.71	-18.95	0.22	-18.07	2.63
**120-140 ns**	-21.20	0.24	-15.98	2.57	-20.66	0.18	-16.34	2.79
**140-160 ns**	-22.81	0.19	-21.99	2.66	-19.90	0.19	-22.11	2.63
**160-180 ns**	-23.22	0.20	-24.42	2.82	-19.27	0.21	-13.04	2.48
**180-200 ns**	-21.19	0.18	-22.10	2.59	-19.66	0.23	-13.33	2.62

S4 External and Major Groove Binding

There were four adducts with Γ_0-RuPy initially placed in external position and in the major groove pocket of both AT and CG sequences, which did not reach a steady state in any of the binding modes considered:

1. Γ_0-RuPy/AT ext
2. Γ_0-RuPy/AT maj
3. Γ_0-RuPy/CG ext
4. Γ_0-RuPy/CG maj

This behaviour was inferred from the RMSD, which showed oscillatory behaviour (related to different geometries of RuPy or relative orientations of the RuPy/DNA adducts) but also abrupt changes in the general trend indicating that the relative position of RuPy with respect to DNA changed.

To further examine the relative position of the RuPy complex with respect to the DNA the number of short and long range N_{SRC} and N_{LRC} were plotted along the equilibration (10 ns) and production (50 ns) stages of each simulation (Figures S1 and S2). N_{SRC} and N_{LRC} are correlated to van der Waals and electrostatic interactions between RuPy and the DNA, respectively. When both N_{SRC} and N_{LRC} were zero, it means that RuPy evolved to the bulk solvent. Intervals with N_{SRC} values below 150 and N_{LRC} non-zero represent situations in which RuPy interacts with the oligonucleotide externally, while higher values of N_{SRC} indicates that RuPy was in one of the groove pockets. Most initial set ups lead to intermittent interaction of RuPy with the DNA sequence. RuPy quickly changed to external binding mode and went to the bulk solvent, sometimes returning to external interaction with different relative orientation.

Figure S1a shows that 4 ns after initially placing Γ_0-RuPy in the major groove of the AT oligonucleotide, it evolved to external binding mode and interacted with the DNA through electrostatic interactions for 10 ns. Then, RuPy approached the major groove pocket and van der Waals interactions took place for approximately 10 ns reflected in the increase of N_{SRC}. Afterwards, RuPy evolved into the bulk solvent evidenced by the absence of contacts. Close to the end of the simulation, RuPy approached the oligonucleotide again and external electrostatic interactions took place as long range contacts appeared. Small van der Waals energy was also present indicating weak interaction with the groove. A similar behaviour was observed for Γ_0-RuPy initially placed

interacting externally with CG (Figure S2b), but in this case when RuPy evolved into the bulk solvent without further interaction with the DNA double strand after 30 ns.

Due to non-convergence of the binding mode in any of the simulations, further binding free energies analysis to characterize the strength of the interaction such as with the MM-PBSA approach could not be performed. Therefore, only a qualitative assessment of the weakness of stability of RuPy/DNA adducts when placed in the major groove pocket or holding external electrostatic interactions was carried out.

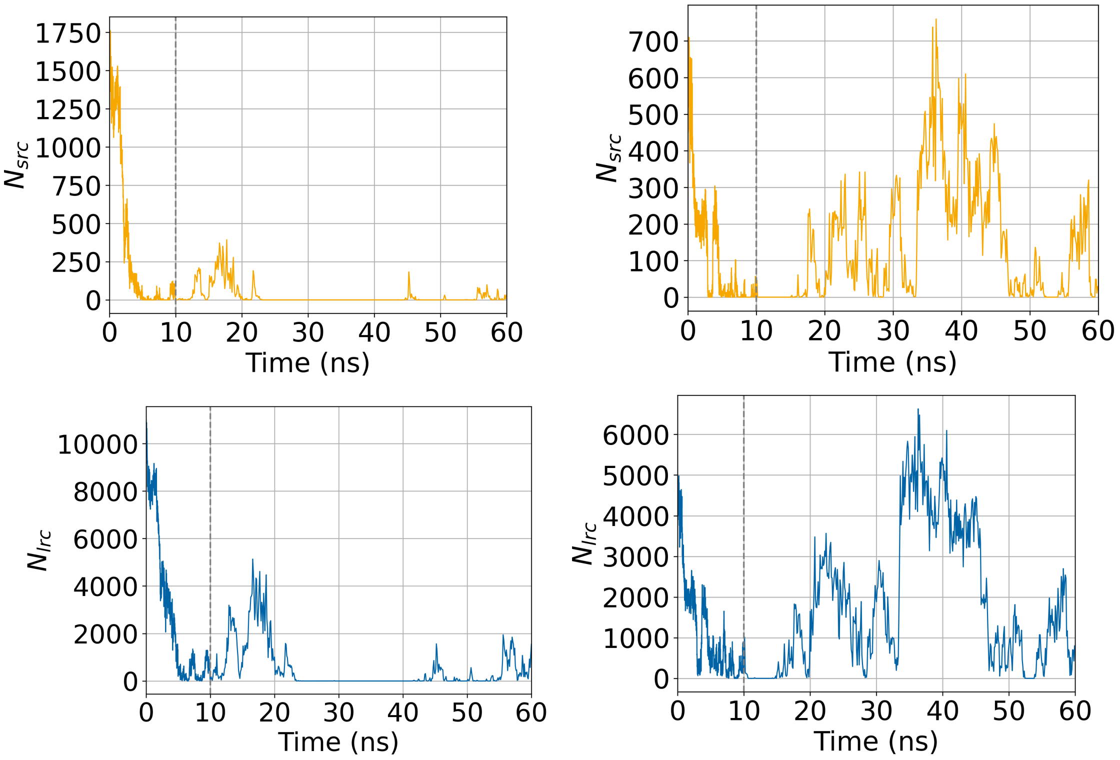

(a) Γ_0-RuPy/AT maj

(b) Γ_0-RuPy/AT ext

Figure S1: Time evolution of the number of short range contacts N_{SRC} (yellow) and long range contacts N_{LRC} (blue) along the equilibration and production runs for (a) Γ_0-RuPy/poly(dA)poly(dT) adduct with major groove initial position and (b) Γ_0-RuPy/poly(dA)poly(dT) adduct initially interacting externally. Vertical line indicates end of equilibration and starting of production run.

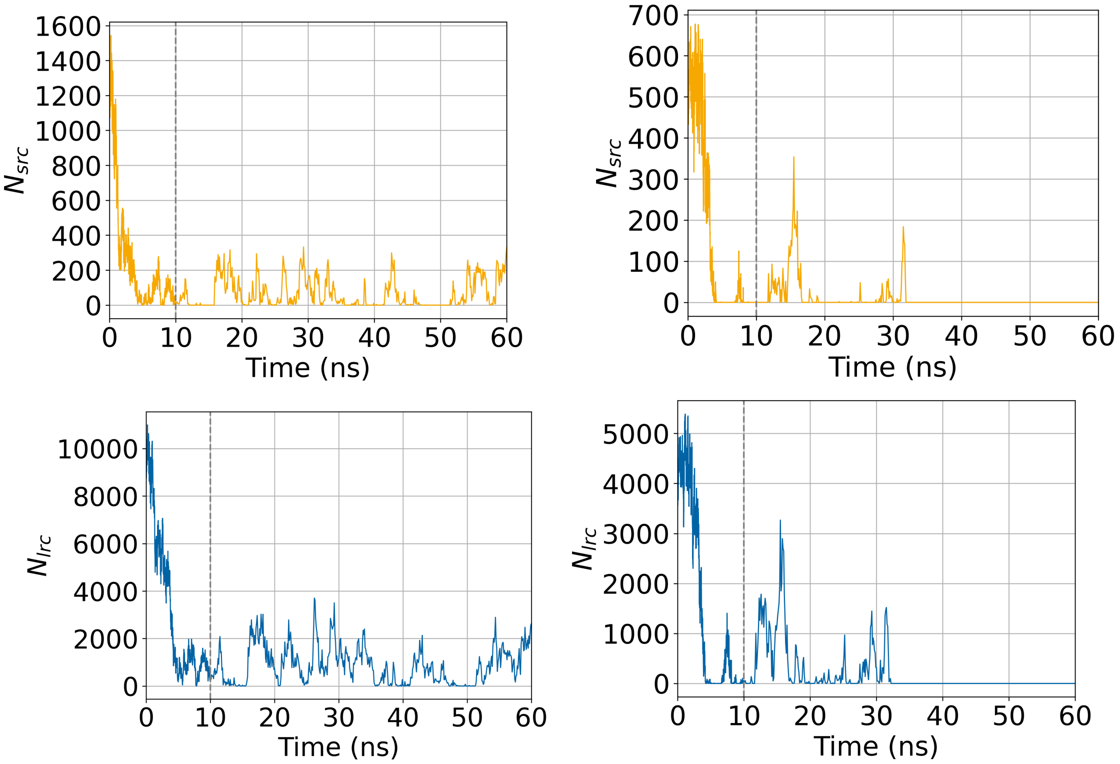

(a) Γ_0-RuPy/CG maj

(b) Γ_0-RuPy/CG ext

Figure S2: Time evolution of the number of short range contacts N_{SRC} in yellow and long range contacts N_{LRC} in blue along the equilibration and production runs for (a) Γ_0-RuPy/poly(dC)poly(dG) adduct with major groove initial position and (b) Γ_0-RuPy/poly(dC)poly(dG) adduct initially interacting externally. Vertical line indicates end of equilibration and starting of production run.

S5 Angle θ Evolution

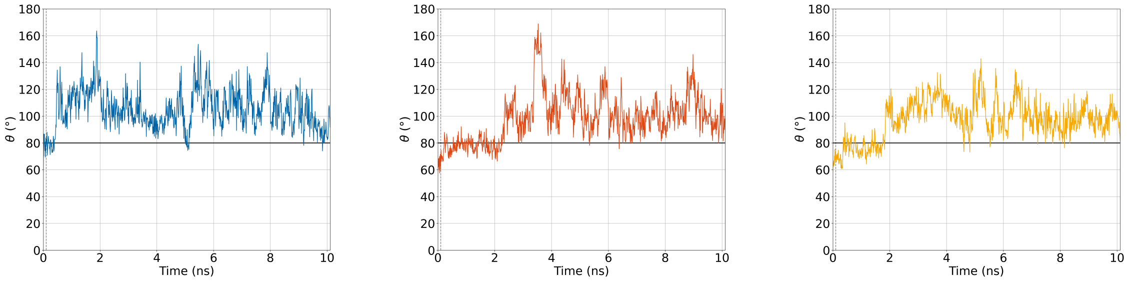

(a) Λ_0-RuPy/AT ext (b) Λ_0-RuPy/AT maj (c) Λ_0-RuPy/AT min

Figure S3: Evolution of angle θ along the heating and equilibration stages for initial Λ_0-RuPy/AT adducts in different initial binding mode.

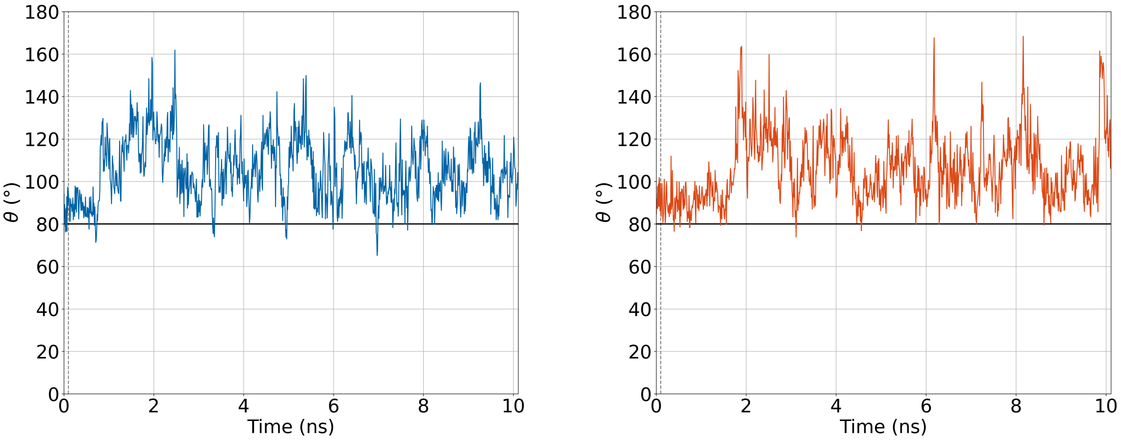

(a) Γ_0-RuPy/AT ext (b) Γ_0-RuPy/AT maj

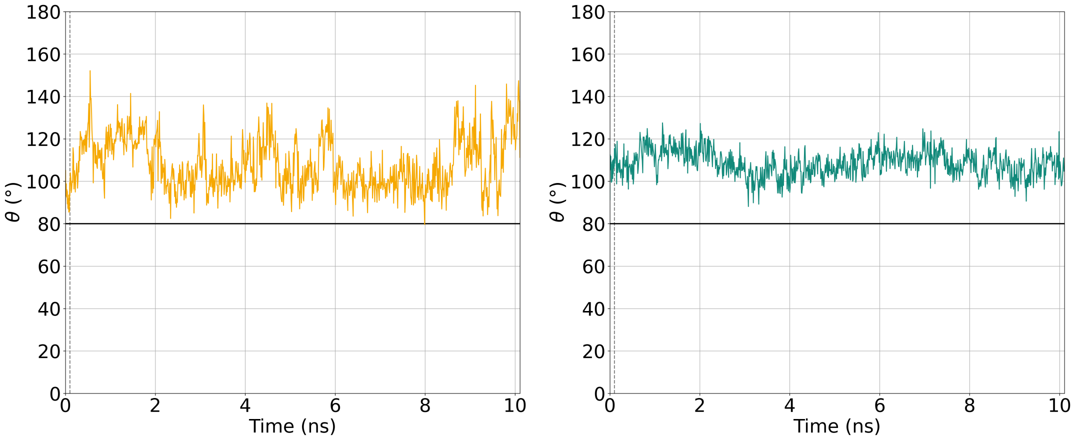

(c) Γ_0-RuPy/AT min (d) Γ_0-RuPy/AT int

Figure S4: Evolution of angle θ along the heating and equilibration stages for initial Γ_0-RuPy/AT adducts in different initial binding mode.

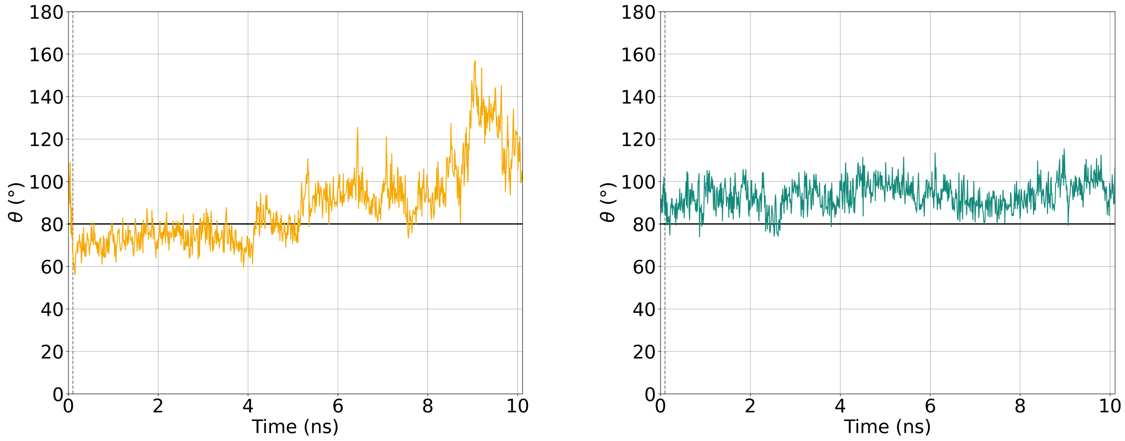

(a) Γ_1-RuPy/AT min

(b) Γ_1-RuPy/AT int

Figure S5: Evolution of angle θ along the heating and equilibration stages for initial Γ_1-RuPy/AT adducts in different initial binding mode.

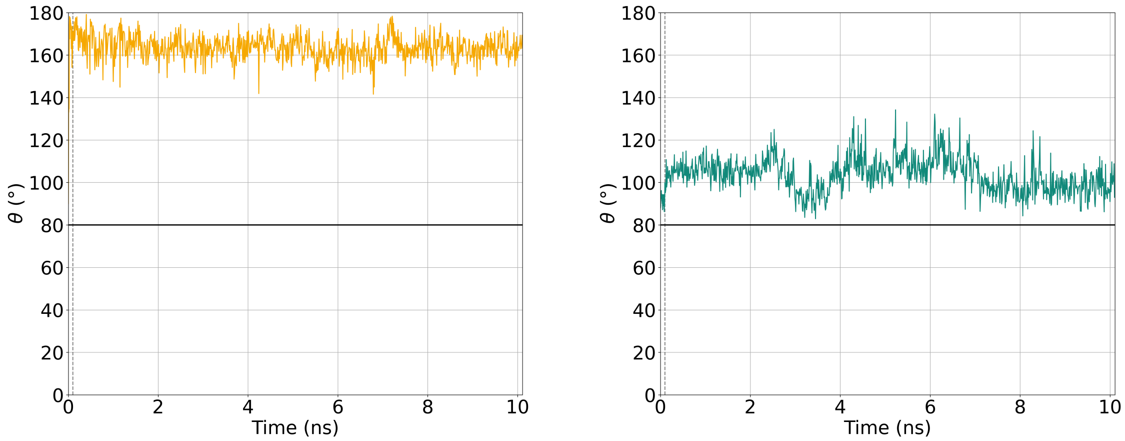

(a) Γ_2-RuPy/AT min

(b) Γ_2-RuPy/AT int

Figure S6: Evolution of angle θ along the heating and equilibration stages for initial Γ_2-RuPy/AT adducts in different initial binding mode.

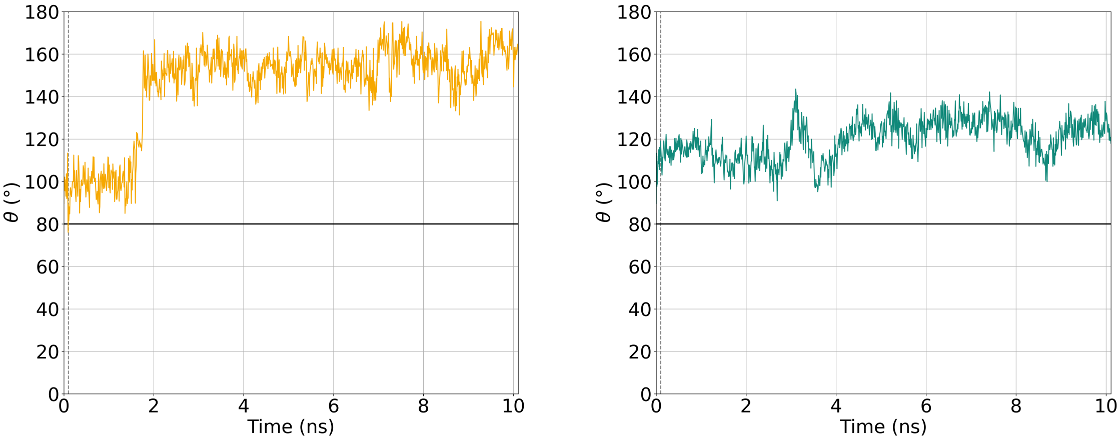

(a) Γ_3-RuPy/AT min (b) Γ_3-RuPy/AT int

Figure S7: Evolution of angle θ along the heating and equilibration stages for initial Γ_3-RuPy/AT adducts in different initial binding mode.

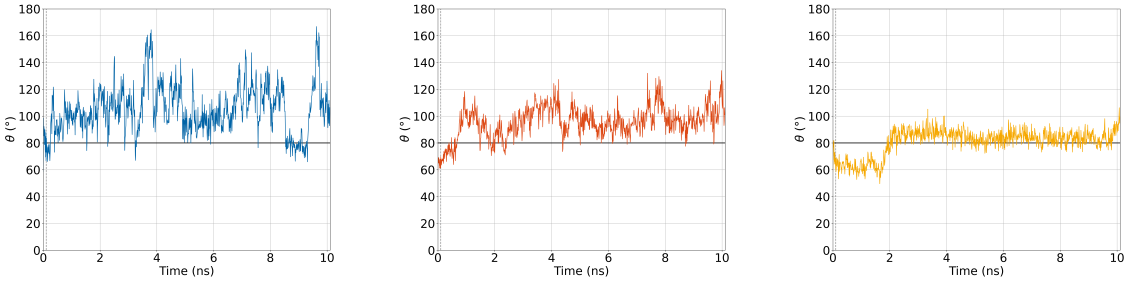

(a) Λ_0-RuPy/CG ext (b) Λ_0-RuPy/CG maj (c) Λ_0-RuPy/CG min

Figure S8: Evolution of angle θ along the heating and equilibration stages for initial Λ_0-RuPy/CG adducts in different initial binding mode.

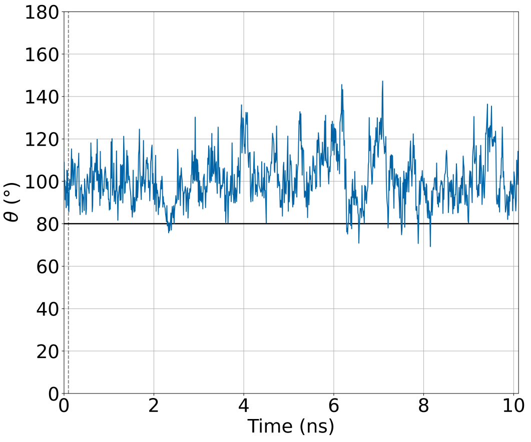

(a) Γ_0-RuPy/CG ext

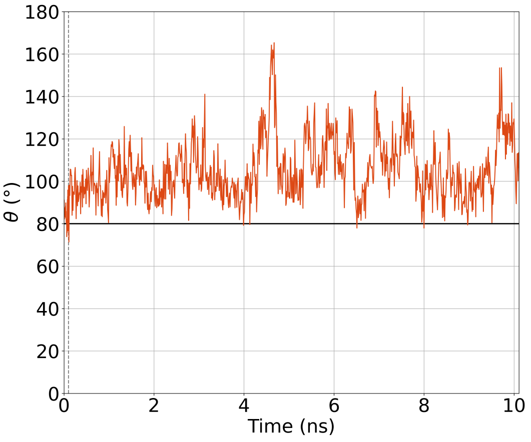

(b) Γ_0-RuPy/CG maj

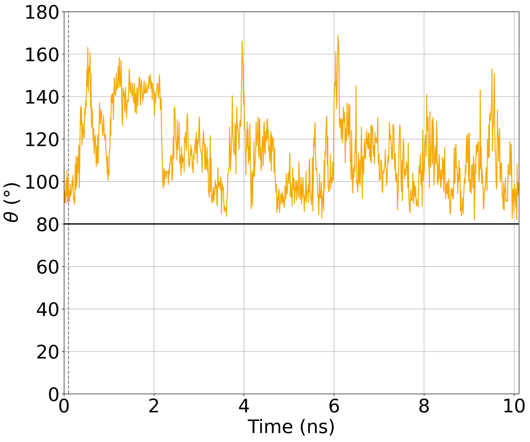

(c) Γ_0-RuPy/CG min

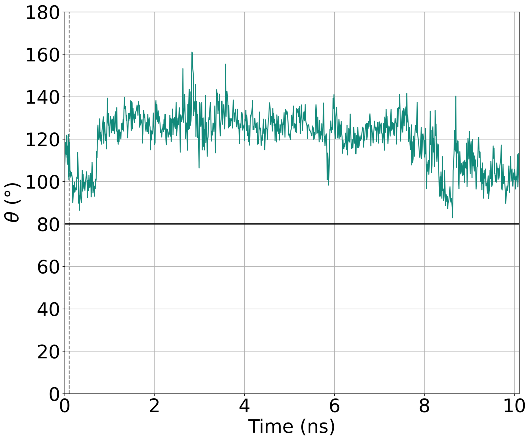

(d) Γ_0-RuPy/CG int

Figure S9: Evolution of angle θ along the heating and equilibration stages for initial Γ_0-RuPy/CG adducts in different initial binding mode.

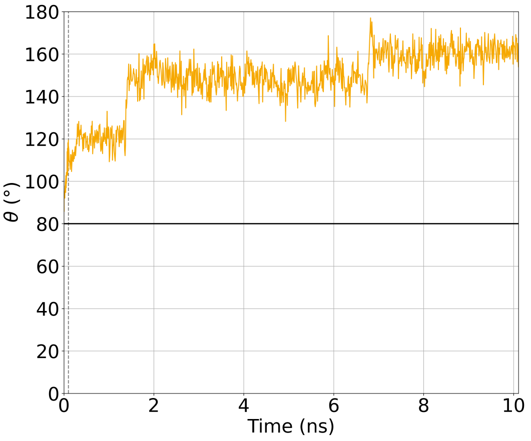

(a) Γ_1-RuPy/CG min

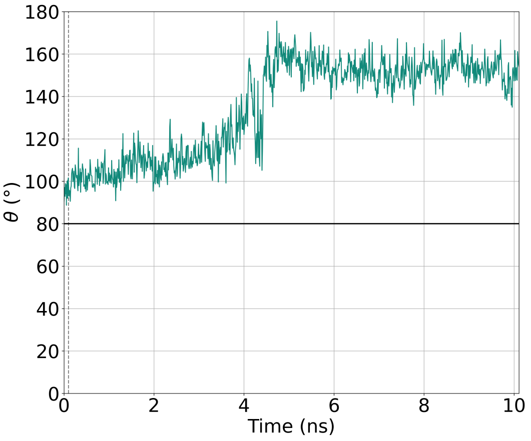

(b) Γ_1-RuPy/CG int

Figure S10: Evolution of angle θ along the heating and equilibration stages for initial Γ_1-RuPy/CG adducts in different initial binding mode.

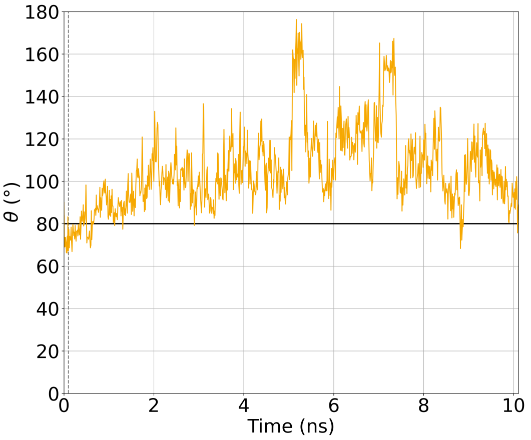

(a) Γ_2-RuPy/CG min

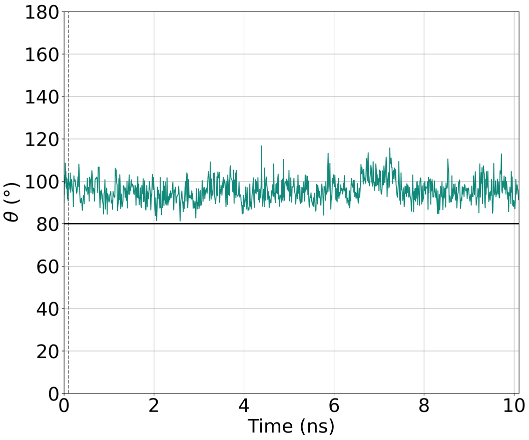

(b) Γ_2-RuPy/CG int

Figure S11: Evolution of angle θ along the heating and equilibration stages for initial Γ_2-RuPy/CG adducts in different initial binding mode.

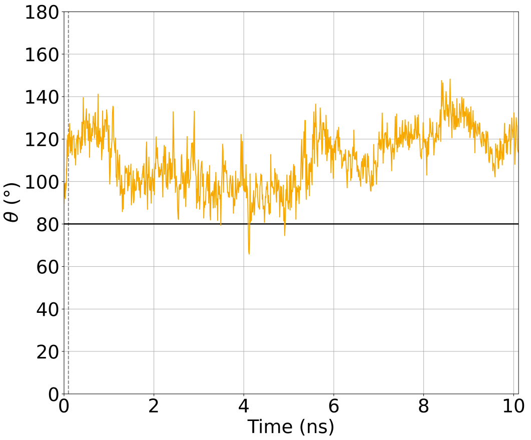

(a) Γ_3-RuPy/CG min

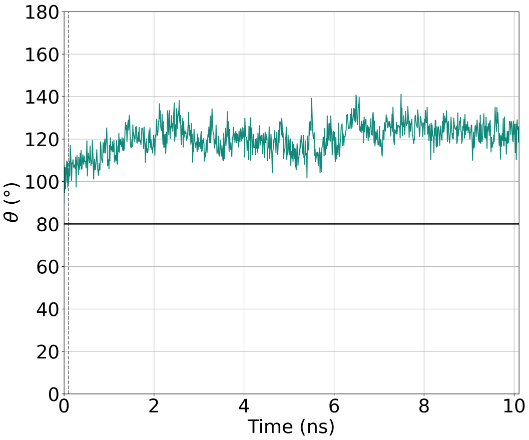

(b) Γ_3-RuPy/CG int

Figure S12: Evolution of angle θ along the heating and equilibration stages for initial Γ_3-RuPy/CG adducts in different initial binding mode.

S6 Torsional Angles Evolution

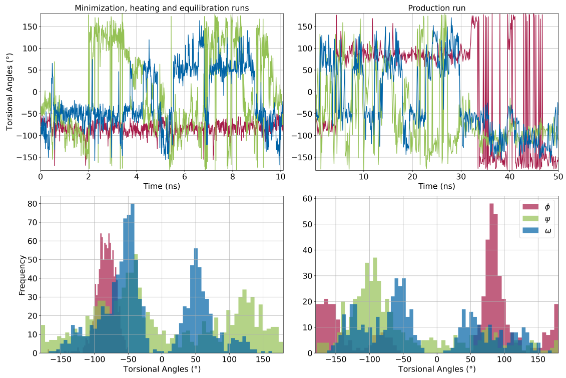

(a) ext

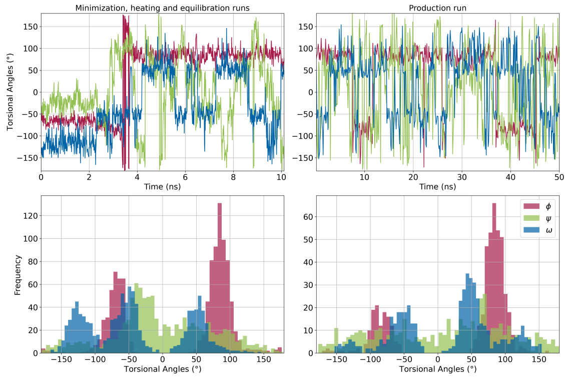

(b) maj

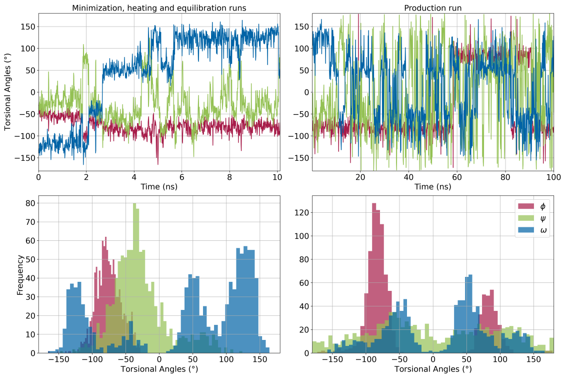

(c) min

Figure S13: Evolution and histograms of torsional angles ϕ, ω and ψ along the minimization, heating and equilibration stages (left) and along the production run (right) for initial Λ_0-RuPy/AT adducts in different initial binding mode.

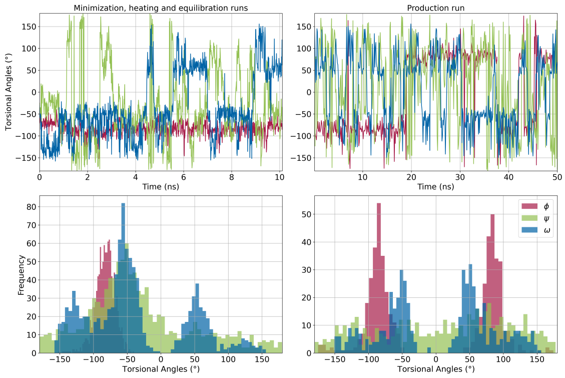

(a) ext

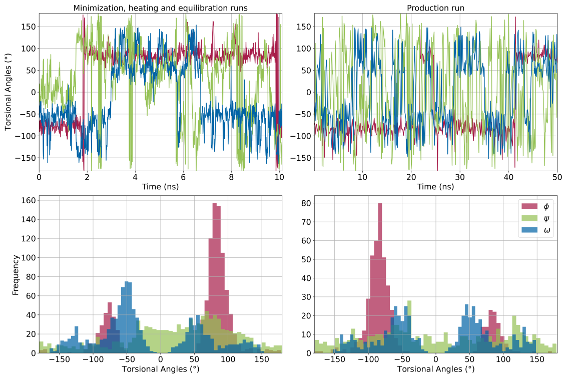

(b) maj

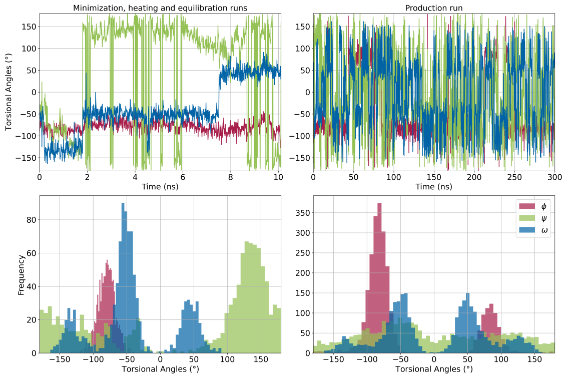

(c) min

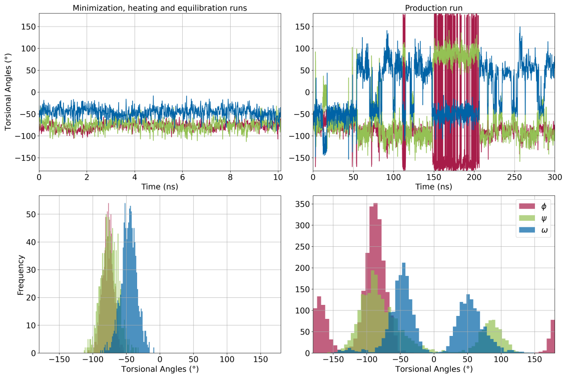

(d) int

Figure S14: Evolution and histograms of torsional angles ϕ, ω and ψ along the minimization, heating and equilibration stages (left) and along the production run (right) for initial Γ_0-RuPy/AT adducts in different initial binding mode.

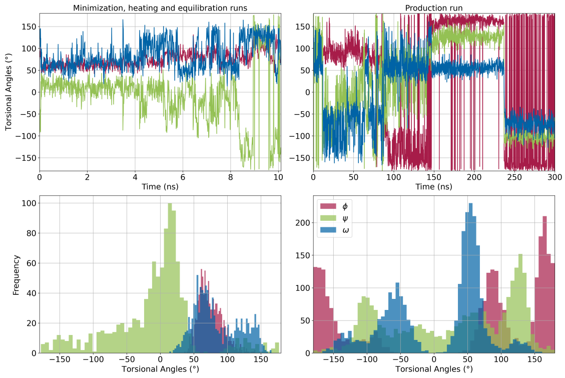

(a) min

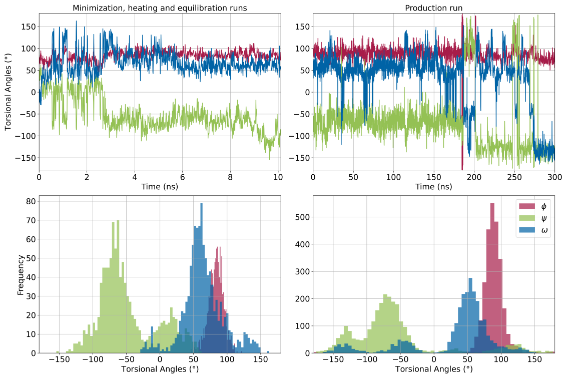

(b) int

Figure S15: Evolution and histograms of torsional angles ϕ, ω and ψ along the minimization, heating and equilibration stages (left) and along the production run (right) for initial Γ_1-RuPy/AT adducts in different initial binding mode.

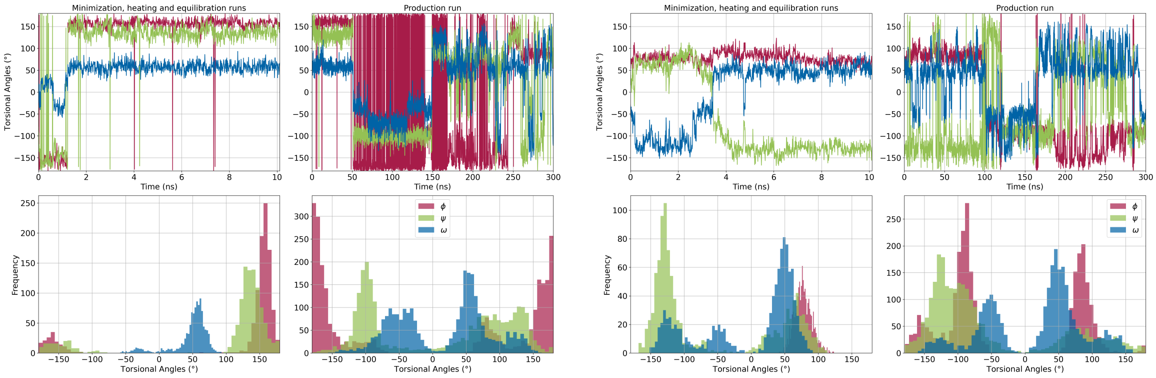

(a) min (b) int

Figure S16: Evolution and histograms of torsional angles ϕ, ω and ψ along the minimization, heating and equilibration stages (left) and along the production run (right) for initial Γ_2-RuPy/AT adducts in different initial binding mode.

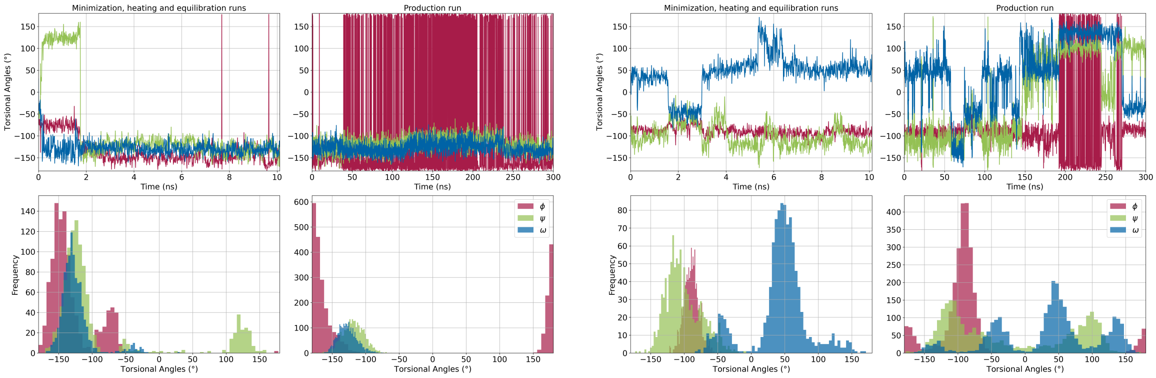

(a) min (b) int

Figure S17: Evolution and histograms of torsional angles ϕ, ω and ψ along the minimization, heating and equilibration stages (left) and along the production run (right) for initial Γ_3-RuPy/AT adducts in different initial binding mode.

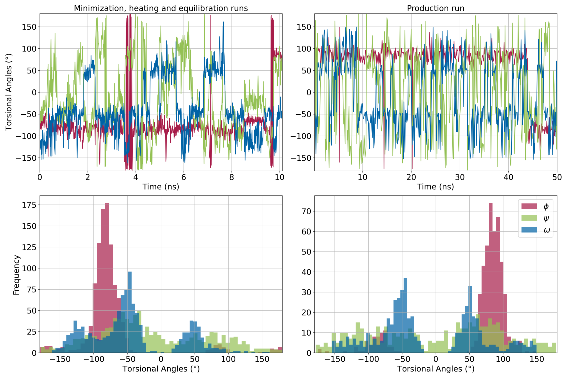

(a) ext

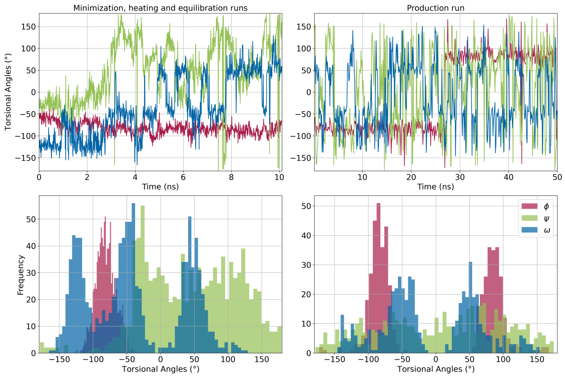

(b) maj

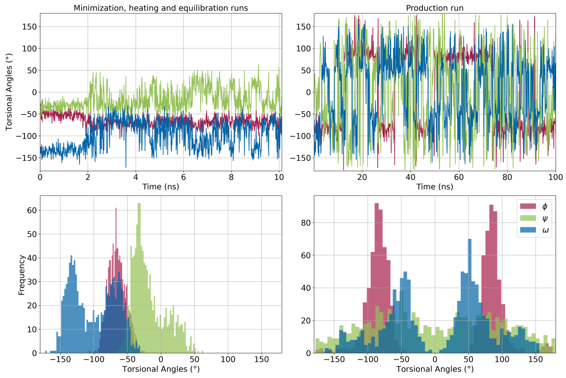

(c) min

Figure S18: Evolution and histograms of torsional angles ϕ, ω and ψ along the minimization, heating and equilibration stages (left) and along the production run (right) for initial Λ_0-RuPy/CG adducts in different initial binding mode.

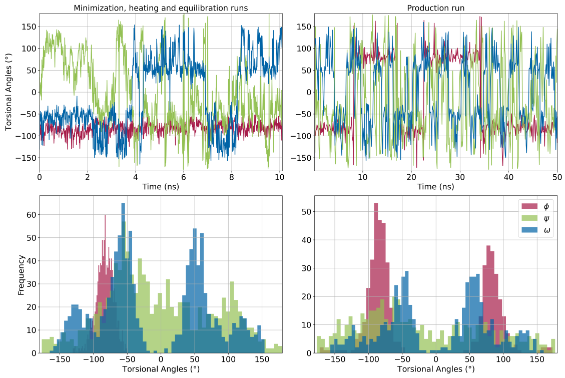

(a) ext

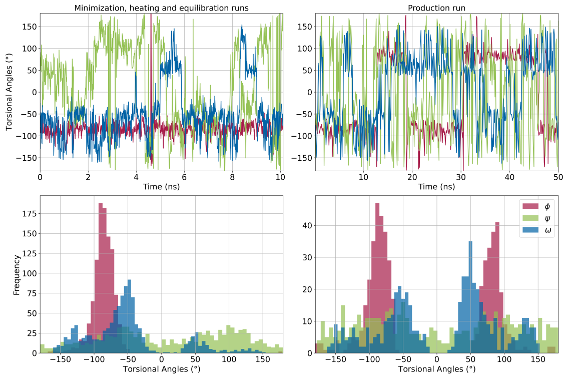

(b) maj

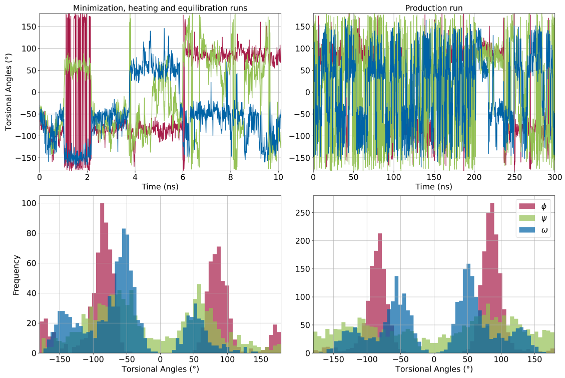

(c) min

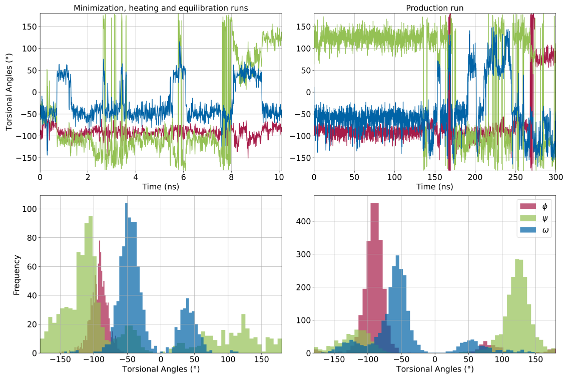

(d) int

Figure S19: Evolution and histograms of torsional angles ϕ, ω and ψ along the minimization, heating and equilibration stages (left) and along the production run (right) for initial Γ_0-RuPy/CG adducts in different initial binding mode.

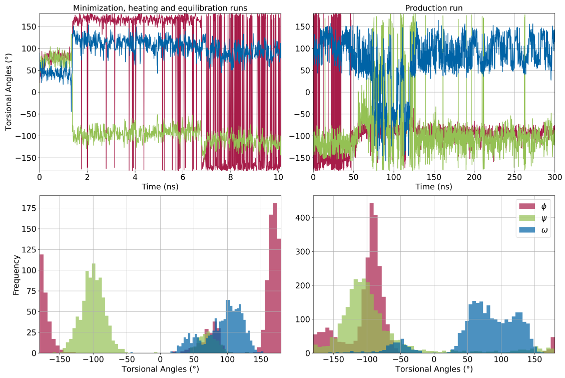

(a) min

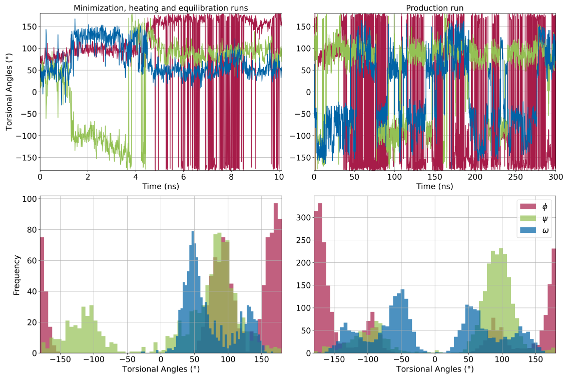

(b) int

Figure S20: Evolution and histograms of torsional angles ϕ, ω and ψ along the minimization, heating and equilibration stages (left) and along the production run (right) for initial Γ_1-RuPy/CG adducts in different initial binding mode.

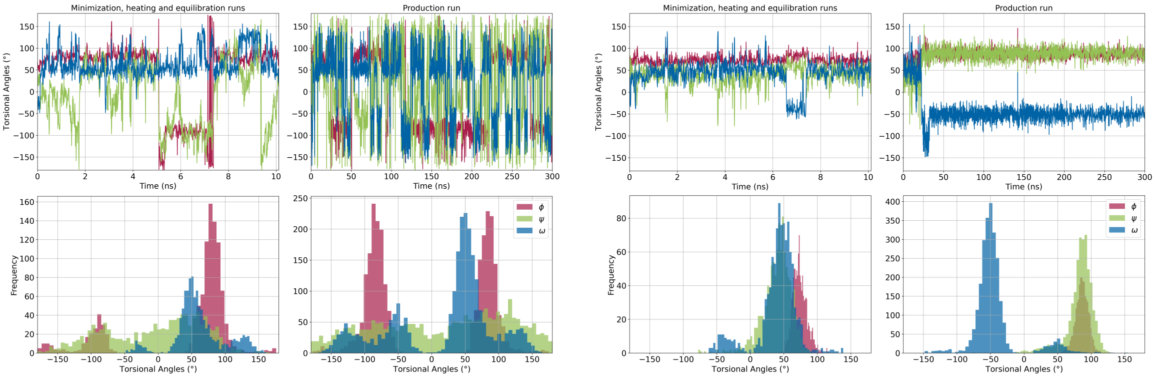

(a) min

(b) int

Figure S21: Evolution and histograms of torsional angles ϕ, ω and ψ along the minimization, heating and equilibration stages (left) and along the production run (right) for initial Γ_2-RuPy/CG adducts in different initial binding mode.

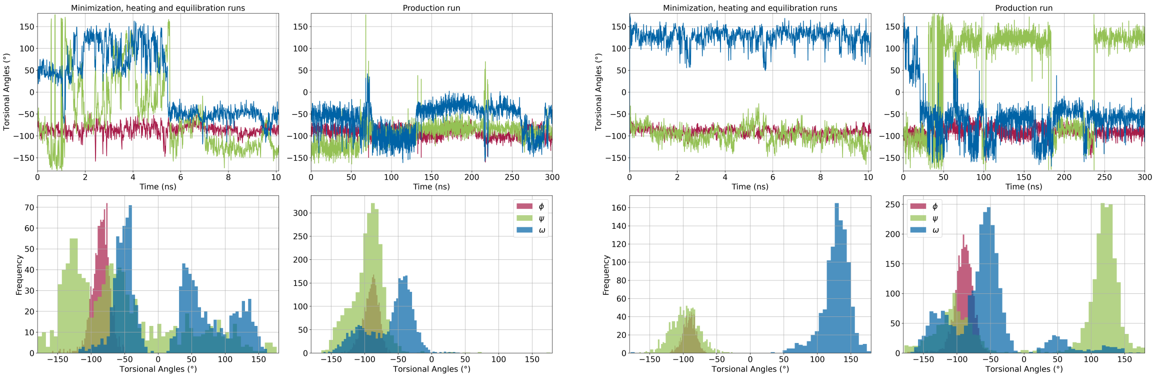

(a) min

(b) int

Figure S22: Evolution and histograms of torsional angles ϕ, ω and ψ along the minimization, heating and equilibration stages (left) and along the production run (right) for initial Γ_3-RuPy/CG adducts in different initial binding mode.

S7 RuPy/CATG

For further analysis, we also generated a mixed DNA sequence: poly(dCATG) ("CATGCATG-CATGCATGCATG") and repeated the calculations. The procedure for building the mixed DNA sequence was the same as for the AT and CG sequences. We selected the Γ_1-RuPy conformation and placed it in several configurations with the poly(dCATG) sequence: intercalated between three different steps—AT-TA, CG-GC, and TG-AC—and in the minor groove pocket, as shown in Figure S23.

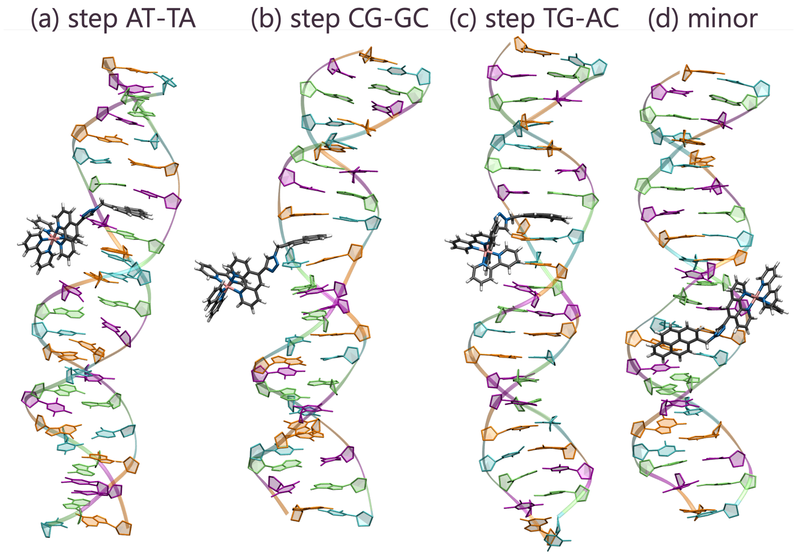

Figure S23: Initial structures of RuPy/DNA adducts. Γ_1-RuPy conformations were initially intercalated between the AT-TA, CG-GC, and TG-AC steps of the poly(dCATG) DNA double strand as well as bounded to the minor groove pocket. Color code for atoms in RuPy and residues in DNA: grey for C, blue for N, pink for Ru, white for H, green for adenine, purple for thymine, light blue for cytosine, and orange for guanine.

The protocol used to generate the MD trajectories was the one described in Section 2.2 of the manuscript. The length of the production runs were 250 ns for the intercalative binding mode and 200 ns for the minor groove binding. All adducts proved to be stable. Specifically, in the three intercalated simulations, the pyrene moiety remained stably intercalated between the chosen nucleobase pairs throughout the entire production run. The remainder of RuPy (the ruthenium atom with its attached ligands) had more freedom, and in all cases experienced a "flip" of orientation with respect to the DNA axis, but the intercalative interaction was consistently maintained. In the minor groove binding simulation, the RuPy complex remained stably bound in the minor groove, but its specific orientation with respect to the DNA underwent frequent variations.

Relative binding free energies were calculated using the MM-PBSA single trajectory protocol (STP), and the results are presented in Table S2 below. The binding free energies for intercalative binding in the mixed sequence (poly(dCATG)) are comparable to those obtained for the poly(dA)poly(dT) and poly(dC)poly(dG) sequences (Tables 3 and 4 of the manuscript). For poly(dCATG), the intercalative binding energies ranged from -21.27 to -21.85 kcal/mol, with similar standard errors across the simulations. In comparison, for the AT sequence (poly(dA)poly(dT)), the intercalative binding energies for Γ_1-RuPy ranged from -18.61 to -24.91 kcal/mol, while for the CG sequence (poly(dC)poly(dG)), the energies ranged from -20.12 to -22.08 kcal/mol. The binding energies obtained for the mixed sequence are well within this range, indicating a comparable stability of intercalative binding for mixed base pairs. For minor groove binding, the binding free

Table S2: Binding free energies in kcal/mol for Γ_1-RuPy geometry initially placed in the minor groove and intercalated between nucleobase pairs of poly(dCATG) DNA double strand calculated with the MM-PBSA approach with the single trajectory protocol (STP).

**Initial Geometry**	**STP**	**SE**
int step AT-TA	-21.85	0.06
int step CG-GC	-21.41	0.06
int step TG-AC	-21.27	0.05
min	-7.45	0.11

energy for the mixed sequence (poly(dCATG)) was -7.45 kcal/mol with a standard error of 0.11, which is slightly less favorable compared to minor groove binding in the AT and CG sequences. The binding free energy for poly(dA)poly(dT) was -8.05 kcal/mol, while for poly(dC)poly(dG), it ranged from -10.16 to -14.76 kcal/mol. This suggests that the mixed sequence shows slightly weaker minor groove binding compared to the homogeneous AT or CG sequences.

The comparison of binding energies between the mixed and homogeneous sequences suggests that in intercalative binding the pyrene moiety of RuPy remains effectively intercalated regardless of the DNA sequence, whether homogeneous or mixed. The binding free energies in all sequences lie within the same range, indicating that intercalative binding is largely sequence-independent in terms of stability. The binding free energies for minor groove binding indicate that the minor groove interaction with RuPy in mixed sequences can be slightly less favorable compared to homogeneous sequences. As anticipated, Ru complex remains effectively intercalated regardless of the DNA sequence, whether homogeneous or mixed. These results further validate our conclusion that the intercalative binding mode is highly stable, and minor groove binding is less stable and presents variability.

References

(1) Galindo-Murillo, R.; Cheatham Thomas E, I. *Nucleic Acids Res.* **2021**, *49*, 3735–3747.

(2) Hou, T.; Yu, R. *Journal of Medicinal Chemistry* **2007**, *50*, PMID: 17300185, 1177–1188.
